# Host-induced aneuploidy and phenotypic diversification in the Sudden Oak Death pathogen *Phytophthora ramorum*

**DOI:** 10.1186/s12864-016-2717-z

**Published:** 2016-05-20

**Authors:** Takao Kasuga, Mai Bui, Elizabeth Bernhardt, Tedmund Swiecki, Kamyar Aram, Liliana M. Cano, Joan Webber, Clive Brasier, Caroline Press, Niklaus J. Grünwald, David M. Rizzo, Matteo Garbelotto

**Affiliations:** Crops Pathology and Genetics Research Unit, USDA Agricultural Research Service, Davis, California 95616 USA; Phytosphere Research, Vacaville, California 95687 USA; Department of Plant Pathology, University of California, Davis, California 95616 USA; Department of Plant Pathology, University of Florida, IFAS, Indian River Research and Education Center, Fort Pierce, Florida 34945 USA; Forest Research, Farnham, Surrey GU10 4LH UK; Horticultural Crops Research Laboratory, USDA Agricultural Research Service, Corvallis, Oregon 97330 USA; Department of Environmental Science, Policy, and Management, University of California, Berkeley, California 947020 USA

**Keywords:** Invasive pathogens, Aneuploidy, Transposable elements, Loss of heterozygosity

## Abstract

**Background:**

Aneuploidy can result in significant phenotypic changes, which can sometimes be selectively advantageous. For example, aneuploidy confers resistance to antifungal drugs in human pathogenic fungi. Aneuploidy has also been observed in invasive fungal and oomycete plant pathogens in the field. Environments conducive to the generation of aneuploids, the underlying genetic mechanisms, and the contribution of aneuploidy to invasiveness are underexplored. We studied phenotypic diversification and associated genome changes in *Phytophthora ramorum,* a highly destructive oomycete pathogen with a wide host-range that causes Sudden Oak Death in western North America and Sudden Larch Death in the UK. Introduced populations of the pathogen are exclusively clonal. In California, oak (*Quercus* spp*.*) isolates obtained from trunk cankers frequently exhibit host-dependent, atypical phenotypes called non-wild type (*nwt*), apparently without any host-associated population differentiation. Based on a large survey of genotypes from different hosts, we previously hypothesized that the environment in oak cankers may be responsible for the observed phenotypic diversification in *P. ramorum*.

**Results:**

We show that both normal wild type (*wt*) and *nwt* phenotypes were obtained when *wt P. ramorum* isolates from the foliar host *California bay* (*Umbellularia californica*) were re-isolated from cankers of artificially-inoculated canyon live oak (*Q. chrysolepis*). We also found comparable *nwt* phenotypes in *P. ramorum* isolates from a bark canker of Lawson cypress (*Chamaecyparis lawsoniana*) in the UK; previously *nwt* was not known to occur in this pathogen population. High-throughput sequencing-based analyses identified major genomic alterations including partial aneuploidy and copy-neutral loss of heterozygosity predominantly in *nwt* isolates. Chromosomal breakpoints were located at or near transposons.

**Conclusion:**

This work demonstrates that major genome alterations of a pathogen can be induced by its host species. This is an undocumented type of plant-microbe interaction, and its contribution to pathogen evolution is yet to be investigated, but one of the potential collateral effects of *nwt* phenotypes may be host survival.

**Electronic supplementary material:**

The online version of this article (doi:10.1186/s12864-016-2717-z) contains supplementary material, which is available to authorized users.

## Background

A growing number of studies have shown that non-native species are able to evolve rapidly following introduction into a new region (reviewed in [1]). Introduced species may be subject to “episodic selection”, i.e. rapid genetic and phenotypic changes possibly leading to incipient speciation caused by sudden and intense environmental disturbance [2]. The concept of “accelerated evolution” may involve two processes: rapid phenotypic change of a particular trait due to differential selective pressure on existing genetic variation, or the generation of *de novo* phenotypic variation in environments where the organism is poorly adapted [3]. *De novo* mutation and mitotic recombination probably account for the rapid phenotypic diversification observed in many introduced fungal pathogens lacking initial genetic diversity, including oomycetes [4, 5].

To date, most mechanisms responsible for *de novo* mutation remain elusive. Aneuploidy, the loss or gain of chromosomes in a nucleus, is frequently generated and can create substantial variation in gene expression by effectively altering gene dosage. Aneuploidy is thus a potentially effective and widespread mechanism for developing novel phenotypic variation in a clonal population [6–8]. For example, antifungal drug resistance in several human pathogenic fungi has been attributed to aneuploidy [9].

In this study, we employed high throughput sequencing to characterize genomic diversity underlying rapid phenotypic diversification in the clonal organism *Phytophthora ramorum. P. ramorum* is the exotic oomycete pathogen responsible for Sudden Oak Death in North America [10], Sudden Larch Death in the UK and Ireland [11], and Ramorum Blight in the nursery trade both in North America and Europe [12, 13]. Its origin is still unknown, but four distinct clonal lineages have been identified. The lineages are believed to have originated from distinct genetically isolated populations in their (presently unknown) region of origin [14]. The NA1, NA2, and to a lesser degree the EU1 lineages are present in North American plant nurseries, while only the EU1 and EU2 lineages are found in Europe [15–17]. Only the NA1 lineage has escaped into natural forest settings on the West Coast of California, where it causes the disease known as Sudden Oak Death [15, 18]. In oaks, disease is caused by lethal trunk cankers, which do not extend below ground [10].

Despite being clonal, isolates of the NA1 lineage display striking variation in colony morphology and growth rate not readily observed in the other lineages. In culture, wild type (*wt*) *P. ramorum* usually grows as a uniform, roughly circular colony mostly appressed to the culture media [19]. However, some NA1 isolates exhibit irregular, unstable, usually slower growing and “fluffy” colony types, referred to as non-wild type (*nwt*). Some *nwt* isolates become senescent, i.e. they cease to grow upon subculturing. Inoculation experiments have shown that isolates with *nwt* morphology are less aggressive than *wt* isolates [19–21].

Recently, we have demonstrated that *P. ramorum* isolates originating from trunks of oak (*Quercus* spp.) are more likely to show *nwt* and senescent phenotypes than those from foliage of California bay (*Umbellularia californica*) [21]. Transcriptome analysis revealed that de-repression of hundreds of transposable elements (TEs) and down-regulation of Crinkler effector homologs (but no other effector families) were common in oak isolates, yet this expression pattern was never observed in isolates from California bay. Our finding of phenotypic differences between isolates from oak and California bay is significant in the context of Sudden Oak Death. It has been demonstrated that oak is a dead-end host with oak infection initiated only via asexual airborne propagules from nearby foliar hosts, most importantly California bay, which does not develop trunk cankers [22, 23]. Consistent with this view, microsatellite markers did not reveal any genetic subdivision between isolates from California bay and oak hosts [21].

Together these observations lead us to hypothesize that in oak, *P. ramorum* undergoes phenotypic diversification after infection. This process could be termed *host-induced phenotypic diversification* (HIPD) because the observed phenotypic changes are host species-dependent. In our case, HIPD is commonly associated with oak, rarely with tanoak, and is virtually absent in bay or *Rhododendron*. To our knowledge phenomena similar to HIPD have not been reported in the literature. However, the putative *de novo* phenotypic variation observed among oak isolates and their reduced aggressiveness [19–21] could be comparable to the increased accumulation of deleterious mutations reported for zoonotic viruses and intercellular symbiotic bacteria when horizontal transmission between hosts was restricted [24, 25]. In this sense, HIPD could be compatible with the current view that oak is a dead-end host for *P. ramorum*. This view appears reasonable in light of the fact that sporulation of *P. ramorum* from oak bark has yet to be observed.

In this study, we attempt to establish direct evidence of the nature of HIPD and of its genetic basis through the artificial inoculation of NA1 isolates on different hosts. We were able to experimentally recreate HIPD, and subsequently through high throughput sequencing-based methods we demonstrated that aneuploidy was associated with HIPD. Although EU1 isolates of *P. ramorum* lineage have exclusively shown *wt* colony morphology [19], EU1 isolates with *nwt* colony morphology have recently been obtained from a rare host, Lawson cypress (*Chamaecyparis lawsoniana*; also known as Port Orford Cedar). We show that these are due to similar chromosomal rearrangements. The observation of HIPD in two distinct evolutionary lineages of *P. ramorum* further emphasizes its potential biological and epidemiological significance.

## Results

### Scoring for *wt* and *nwt* colony morphology

Some *wt* cultures developed into *nwt* colonies upon subculturing [19]. For example, when a culture of oak isolate Pr-102 showing *wt* morphology was subcultured, 29 out of 100 colonies displayed *nwt* morphology within seven days (Petri plate control in Table 1, Additional file 1). Additionally, some Pr-102 *nwt* cultures also became senescent. In contrast, NA1 isolates from foliar hosts consistently displayed homogenous *wt* colony type as demonstrated by the fact that no *nwt* was observed out of a total of 348 subcultures. *Nwt* morphology was significantly more common in Pr-102 than in isolates from foliar hosts (Fisher’s exact test *p* = 2.2 × 10^−16^). When *nwt* colony morphology was observed upon further subculturing, we assigned the original isolate to the *nwt* phenotype, even if the original isolate did not consistently display *nwt* colony morphology. Thus, *nwt* colony phenotype in this study was defined as the ability to develop the *nwt* morphology upon subculturing. To track this phenomenon, we precisely indicate [21] colony phenotypes and the known history for each isolate in our results (Additional file 2). For example, Pr-1556#7#1 (*nwt*, bay➔oak➔race tube) indicates isolate of Pr-1556#7#1 was originally isolated from a California bay and subsequently passed through oak via an artificial inoculation, and then transferred to, and recovered from a race tube.

**Table 1:** Summary of phenotypic conversion upon passage experiments

Inoculated host, year	# Trees (# Leaves)^a^	Inoculum; duration in host	Phenotypes^f^		Total
			*wt*	*nwt*	
Petri plate control	n/a	Pr-710, Pr-745, Pr-1556, Pr-1557, ND886 (*wt,* foliar hosts)^b^ ; n/a	348	0	348
	n/a	Pr-102 (*nwt*, oak)^c^ ; n/a	71	29	100
Canyon live oak, 2010	8	Pr-710 and Pr-745 (*wt*, bay); 20 weeks	37	25	62
	10	Pr-710 and Pr-745 (*wt*, bay); 40 weeks	23	35	58
Shreve oak, 2010	2	Pr-710 and Pr-745 (*wt*, bay); 20 weeks	6	2	8
Canyon live oak, 2012	4	Pr-1556 and Pr-1557 (*wt*, bay); 20 weeks	27^d^	0	27
California bay, 2012	1 (9)	Pr-745 (*wt*, bay); 20 weeks	9	0	9
California bay, 2013	3 (27)	ND886 (*wt,* camellia); 4 to 25 weeks^e^	20	0	20
	3 (27)	Pr-102 (*nwt*, oak); 4 to 25 weeks^e^	16	2	18

^a^The total number of trees and the total number of leaves re-isolation were attempted. The pathogen was inoculated on oak stems or California bay leaves

^b^colony morphology of five *wt* isolates grown on Petri plates was scored. 24, 24, 100, 100 and 100 replicates were made for Pr-710, Pr-745, Pr-1556, Pr-1557, and ND886, respectively

^c^Pr-102 displaying *wt* colony morphology was used as inoculum. The 71 subcultures showing *wt* morphology were expected to display *nwt* colony morphology at a certain percentage upon subsequent subculturing

^d^Most of the tested *wt* isolates displayed *nwt* after passage through race tubes

^e^re-isolation was made 4, 12 and 25 weeks post-inoculation

^f^Criteria for scoring of *wt/nwt* are described in Evaluation of colony morphology in Methods

### Experimental generation of phenotypes associated with *oak* isolates

**Fig. 1:**
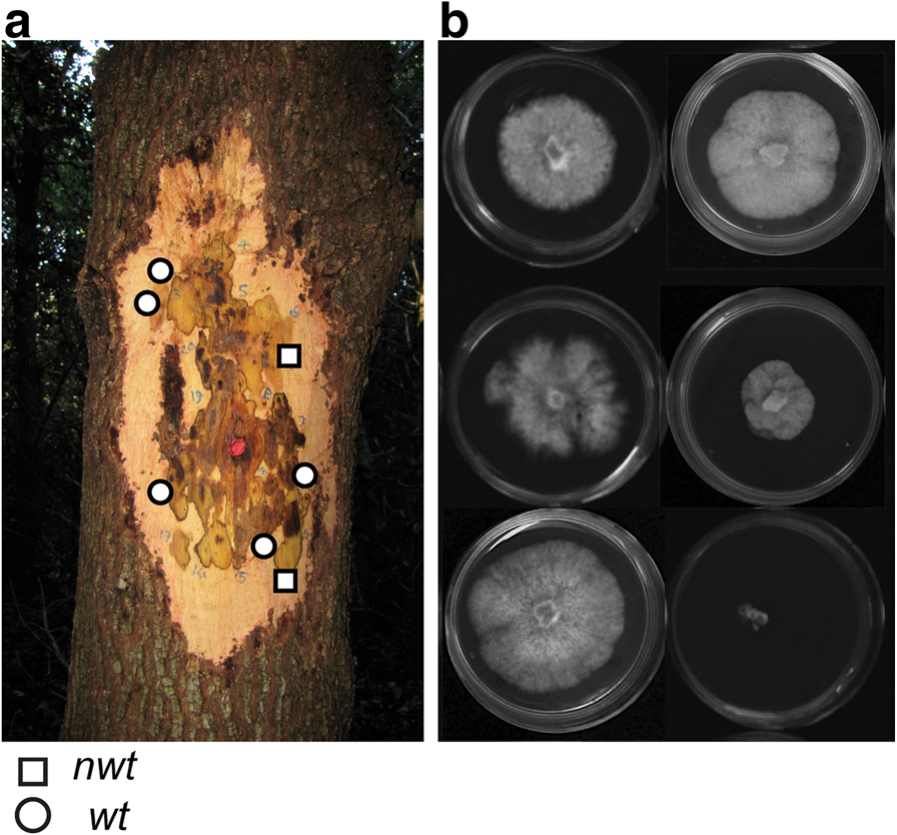
*Nwt* colony phenotype was experimentally reproducible by oak passage experiment. **a** Scraping off the outer bark 20 weeks post-inoculation revealed diseased tissue. The red mark at the center indicates the inoculation point. Isolates recovered from circle and square marks yielded *wt* and *nwt* colonies, respectively. **b** Isolates show diverse colony morphology. Top left: Pr-745 (*wt*, bay), top right: Pr-710 (*wt*, bay), both are wild type isolates used as inocula. Middle left and right: examples of re-isolates from canyon live oak showing *nwt* colony morphology. Bottom left: a re-isolate from canyon live oak showing a *wt* colony morphology, bottom right: an example of early senescence

We hypothesized that the oak host environment in which *P. ramorum* grows induces HIPD. In order to experimentally demonstrate HIPD, phenotypic changes during host passage experiments were monitored. Two passage experiments were conducted on oak using four California bay isolates as inocula. In the first passage experiment, two *wt P. ramorum* isolates, Pr-710 (*wt*, bay) and Pr-745 (*wt*, bay - isolated from rainwater adjacent to infected California bays), were inoculated into stems of mature canyon live oak (*Q. chrysolepis*) and Shreve oak (*Q. parvula* var. *shrevei*) in July 2010 [26]. In December 2010 and April 2011, 20 and 40 weeks post-inoculation, respectively, the pathogen was re-isolated from lesions that had formed in the phloem and underlying xylem (Fig. 1). These are termed “re-isolates” hereafter. Thirty-nine percent of re-isolates collected 20 weeks post-inoculation (of 70 from canyon live oak and Shreve oak combined) and 60 % collected 40 weeks (of 58 from canyon live oak only) were assigned to *nwt* phenotype. In contrast, none of 48 in vitro subcultures of isolates Pr-710 and Pr-745 displayed *nwt* phenotype (Petri plate control in Table 1).

In order to obtain baseline phenotypic conversion rate in foliar hosts, California bay leaves were inoculated with two *wt* isolates, Pr-745 (*wt*, bay) and ND886 (*wt*, camellia), and re-isolate phenotypes were evaluated. None of the 29 re-isolates derived over 25 weeks from Pr-745 and ND886 inoculations showed *nwt* morphology. Together this series of passage experiments indicate that canyon live oak and Shreve oak, but not California bay, induced phenotypic conversion from *wt* to *nwt*; therefore, we conclude that HIPD is experimentally reproducible. In addition, the *nwt* isolate Pr102 was passed through California bay to evaluate the potential reversibility of *nwt* colony phenotype by epigenetics or phenotypic plasticity. However, we failed to detect the reversibility with certainty: two out of 18 (11 %) re-isolates derived from Pr102 displayed *nwt* colony morphology, which did not differ significantly (Fisher’s exact test *p* = 0.3) from the rate of *nwt* morphology observed for in vitro subcultures (29 *nwt* out of 100, Table 1).

The oak inoculation experiment was repeated with different isolates, Pr-1556 and Pr-1557 (*wt*, bay) in July 2012. In December 2012 (20 weeks post-inoculation), 27 axenic cultures of Pr-1556 and Pr-1557 were re-isolated from phloem tissue of resulting cankers (Table 1). Contrary to the first passage experiment, no isolates with *nwt* morphology or senescence were observed at the moment they were cultured out of the hosts in which they were inoculated. However, punctuated growth patterns, one of the common characteristics of the *nwt* phenotype, were observed 10 to 16 weeks after they were placed in race tubes and monitored (see below).

### Validation of experimentally induced phenotypic conversion by means of global mRNA profiling

**Fig. 2:**
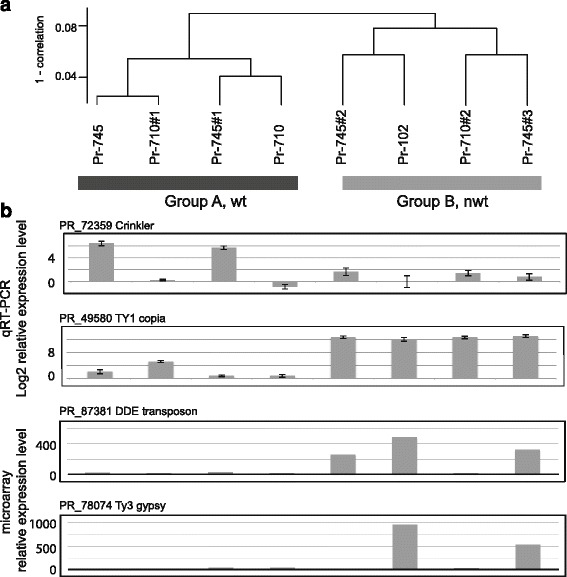
Transcriptome analyses support host-induced phenotypic diversification. **a** Eight cDNA samples from Petri plate cultures were clustered based on their global expression patterns of 14,339 transcripts. Group A consists of the two California bay isolates used as inocula and re-isolates from canyon live oak, all having *wt* colony phenotype. Isolates in group B are either from naturally infected coast live oak or from artificially-inoculated canyon live oak, and all have *nwt* colony phenotype. **b** Examples of mRNA profiles of *P. ramorum* genes differentially expressed between group A and group B. The order of isolates is same as that in **a**. A gene model number and its annotation are shown for each profile. Gene expression of PR_72359 and PR_49580 were estimated by qRT-PCR, and log2 fold changes (−ΔΔCT) are shown. PR_76099 was used as endogenous control gene, and expression levels were standardized to the genome sequence strain Pr-102. For PR_49580, because the reference PR-102 had a high expression level, its expression was offset by 12 for the presentation purpose. Error bars represent SD in technical replicates. For PR_87381 and PR_78074, bars represent estimates of the relative expression levels according to microarray mRNA profiling

Re-isolates and isolates from naturally infected oak with *nwt* phenotype were morphologically indistinguishable. We have previously reported that *wt* and *nwt* isolates show distinct global mRNA patterns [21], indicating physiological differences associated with the colony types. In order to validate the experimental induction of HIPD, we performed global mRNA profiling for the two isolates Pr-710 (*wt*, bay) and Pr-745 (*wt*, bay) used in the oak passage experiment and five re-isolates with *wt* or *nwt* colony types (Fig. 2). Pr-102 (*nwt*, oak), an isolate derived from a naturally infected oak exhibiting a distinct global mRNA expression pattern associated with *nwt* phenotype [21] was included in the analysis as a “*nwt* standard”. Cluster analysis of global mRNA profiles revealed two major groups, each associated with a specific colony phenotype. The two *wt* isolates used in the inoculation trial and two re-isolates showing *wt* phenotype formed one group (Group A in Fig. 2a), while the three re-isolates showing *nwt* phenotype plus Pr-102 formed the other group (Group B). Hence, global mRNA profiling corroborates that isolates with *nwt* phenotype are physiologically equivalent whether obtained directly from naturally infected trees or obtained from artificially inoculated oak. De-repression of transposable elements (TEs), a hallmark of the *nwt* phenotype, was observed in the three *nwt* re-isolates (Fig. 2b). In addition, down-regulation of Crinkler effector homologs, which was common in oak isolates, was observed in two *nwt* re-isolates derived from Pr-745. We have therefore experimentally established that the *nwt* phenotype is generated in oak.

### Re-isolates showed accelerated growth rate in race tubes

**Fig. 3:**
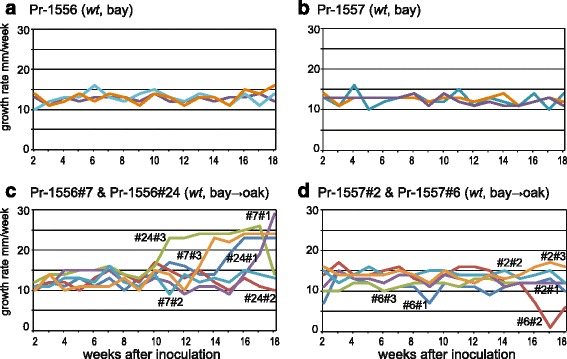
*Wt* re-isolates from oak became differentiated from California bay isolates on race tubes. Growth rates of triplicates of California bay isolates **a** Pr-1556 and **b** Pr-1557 were relatively constant for 18 weeks. Growth rates of triplicates of two re-isolates derived from each of **c** Pr-1556 and **d** Pr-1557 were also relatively constant in the first eight weeks (growth rates of the bay isolates and re-isolates are not significantly different between the 2nd and 9th weeks, one-way ANOVA *p* = 1). However, fluctuation in growth rates became apparent after ten weeks in the race tube (between 10 and 18th weeks, growth rates of bay isolates and re-isolates were significantly different, one-way ANOVA *p* = 3.5 × 10^−3^)

None of the 27 Pr-1556 and Pr-1557 (*wt*, bay- > oak) re-isolates obtained in the second oak passage experiment initially had a *nwt* phenotype. Among the re-isolates in the first oak passage experiment, *wt* re-isolates occasionally displayed *nwt* morphology on subsequent subculturing, suggesting *nwt* phenotype can be latent and develop during continued cultivation on artificial media. Although no *nwt* colony morphology was observed among isolates in the second passage experiment, we hypothesized the likelihood of *wt* to *nwt* conversion would be elevated in the re-isolates from oaks. In order to address this possibility, three transfers each were made from four re-isolates from the second oak passage experiment, the two original isolates, and a camellia isolate, onto 40 cm-long race tubes. Growth rates were monitored once a week (Fig. 3). In the course of 18 weeks, Pr-1556 (*wt*, bay) and Pr-1557 (*wt*, bay) displayed consistent growth rates (Fig. 3a, b), while growth rates of *wt* re-isolates from oaks (Fig. 3c, d) accelerated or decelerated considerably after 10 weeks. The accelerated growth rate observed in re-isolates is hereafter referred to as *growth acceleration phenotype* (GAP). In the race tube, the hyphal tips at the growing front do not always extend at the same rate: the fastest-growing hyphal tip can ramify, expand and take over the colony front. In other words, a fast growing trait can be selected when variation in growth rates of individual hyphal tips occurs, which is likely the case for GAP.

All cultures were transferred back to duplicate Petri plates once the mycelial fronts reached the far end of race tubes. In total, three of the four original re-isolates showed *nwt* or senescent colony types in at least one replicate after recovery from the race tubes (Table 2). In contrast, none of the California bay and camellia isolates showed *nwt* phenotypes after recovery from race tubes (Table 2). In summary, the race tube experiment demonstrated that *wt* re-isolates from oak have a greater or latent propensity for the conversion to *nwt* colony morphology than foliar isolates (Fisher’s exact test, *p* = 4.6 × 10^−3^).

**Table 2:** Colony phenotypes after retrieval from race tubes

isolate	replica #1	replica #2	replica #3
Pr-1556 (*wt*, bay)	*wt/wt*	*wt/wt*	*wt/wt*
Pr-1557 (*wt*, bay)	*wt/wt*	*wt/wt*	*wt/wt*
ND886 (*wt*, camellia)	*wt/wt*	*wt/wt*	*wt/wt*
Pr-1556#7 (*wt*, bay- > oak)	*wt/wt*	*wt/nwt*	*wt/nwt*
Pr-1556#24 (*wt*, bay- > oak)	senes./senes.	senes./senes.	*nwt/nwt*
Pr-1557#2 (*wt*, bay- > oak)	*wt/wt*	*wt/wt*	*wt/wt*
Pr-1557#6 (*wt*, bay- > oak)	senes./senes.	*nwt*/senes.	senes./senes.

For each race tube replicate, two pieces of mycelial plugs were retrieved and colony phenotypes; *wt, nwt* or early senescence (senes.), were scored

### Analysis of chromosomal copy number variations (CCNVs)

The results of the inoculation experiments uphold our interpretation of the mechanisms underlying HIPD, implying that the environment in oak cankers triggers *de novo* mutations in *P. ramorum*. As a potential source of the *nwt* phenotype, we examined changes in chromosomal copy number, since these have been reported in association with phenotypic alterations after exposure to antifungal chemicals [6, 7, 9, 27]. A read-depth based method called BIC-seq [28] detected variations in chromosomal copy number (CCNV) among NA1 *P. ramorum* isolates. Remarkably, CCNVs appeared to be colony phenotype dependent. While 17 out of the 19 *wt* NA1 isolates displayed 0–26 regions with CCNVs (sizes between 100 and 31,800 bp), all of the seven isolates with *nwt* colony phenotype, including four oak isolates and three re-isolates from oak, produced 171–903 regions with CCNVs (sizes between 100 and 644,200 bp) (the number of CCNV regions in *nwt* is significantly different from that in *wt*, Fisher’s exact test, *p* = 5.5 × 10^−5^; the size distributions of CCNV regions in *wt* and *nwt* are also significantly different, Mann–Whitney U test, *p* = 5.5 × 10^−12^) (Table 3).

**Table 3:** Summary of SNP and CCNV analyses

Isolate group	CCNV category^a^	Isolate	Colony phenotype	Reference isolate	# CCNV regions (mean size bp)
NA1	Normal Euploid	MK649b (*wt*, bay)	*wt*	used as ref.	n/a
	Normal Euploid	Pr-1556 (*wt*, bay)	*wt*	used as ref.	n/a
	Normal Euploid	Pr-710 (*wt*, bay)	*wt*	used as ref.	n/a
	Normal Euploid	Pr-745 (*wt*, bay)	*wt*	used as ref.	n/a
	Normal Euploid	Pr-745#1 (*wt*, bay- > oak)	*wt*	Pr-710	0 (0)
	Normal Euploid	MK548 (*wt*, bay)	*wt*	MK649b	4 (2,625)
	Normal Euploid	MK79j (*wt*, bay)	*wt*	Pr-710	4 (4,350)
	Normal Euploid	Pr-1556#2 (*wt*, bay- > race tube)	*wt*	Pr-710	6 (2,932)
	Normal Euploid	MK106 (*wt*, oak)	*wt*	MK649b	8 (2,211)
	Normal Euploid	Pr-710#1 (*wt*, bay- > oak)	*wt*	Pr-710	9 (3,580)
	Normal Euploid	MK649a (*wt*, bay)	*wt*	Pr-710	10 (3,732)
	Normal Euploid	ND886_UC#1 (*wt*, camellia- > bay)	*wt*	Pr-1556	10 (2,360)
	Normal Euploid	MK516d (*wt*, oak)	*wt*	Pr-710	11 (2,285)
	Normal Euploid	ND886_QA#7 (*wt*, camellia- > oak)	*wt*	MK649b	11 (3,045)
	Normal Euploid	Pr-1556#7 (*wt*, bay- > oak)	*wt*	MK649b	12 (1,350)
	Normal Euploid	MK558 (*wt*, oak)	*wt*	MK649b	26 (1,050)
	Normal Euploid	ND886_QA#9 (*wt*, camellia- > oak)	*wt*	Pr-1556	20 (5,450)
	2x cnLOH	MK516a (*nwt*, oak)	*nwt*	Pr-1556	287 (3,876)
	1x CCNV	Pr-140.9 (*wt*, oak)	*wt*	Pr-1556	218 (8,598)
	CCNV heterokaryon	Pr-745#4 (*nwt*, bay- > oak)	*nwt*	Pr-1556	171 (15,536)
	CCNV heterokaryon	Pr-140.7 (*nwt*, oak)	*nwt*	Pr-1556	256 (11,375)
	CCNV heterokaryon	Pr-1556#7#1 (*nwt*, bay- > oak- > race tube)	*nwt*	Pr-1556	271 (41,465)
	3x CCNV	Pr-102_UC#4 (*wt*, oak- > bay)	*wt*	Pr-1556	114 (27,238)
	3x CCNV	Pr-102 (*nwt*, oak)	*nwt*	Pr-745	215 (14,358)
	3x CCNV	Pr-745#3 (*nwt*, bay- > oak)	*nwt*	Pr-745	339 (17,271)
	3x CCNV + 2x cnLOH	Pr-16 (*nwt*, oak)	*nwt*	Pr-745	903 (5,924)
CCNV generation^b^	Normal Euploid	Pr-1556 (*wt*, bay)	*wt*	used as ref.	n/a
	Normal Euploid	Pr-1556#2 (*wt*, bay- > race tube)	*wt*	Pr-710	6 (2,932)
	Normal Euploid	Pr-1556#7 (*wt*, bay- > oak)	*wt*	MK649b	12 (1,350)
	CCNV heterokaryon	Pr-1556#7#1 (*nwt*, bay- > oak- > race tube)	*nwt*	Pr-1556	271 (41,465)
EU1^c^	CCNV heterokaryon	P2363 (*wt, C. lawsoniana*), middle of the lesion	*wt*	Pr-1556	2,060 (5,105)
	CCNV heterokaryon	P2346 (*nwt, C. lawsoniana*), bottom of the lesion	*nwt*	P2363	473 (21,500)
	CCNV heterokaryon	P2386 (*nwt, C. lawsoniana*), top of the lesion	*nwt*	P2363	275 (6,546)

^a^According to CCNV and allele ratio analyses, isolates were categorized into five groups. Details are in the main text

^b^NA1 clonal isolates subjected to a series of passage experiments were consolidated to highlight the generation of CCNVs. These isolates are also listed under NA1 isolates

^c^The three EU1 isolates were obtained from the middle, bottom and top of a ca. 4 m long lesion on *Chamaecyparis lawsoniana*

**Fig. 4:**
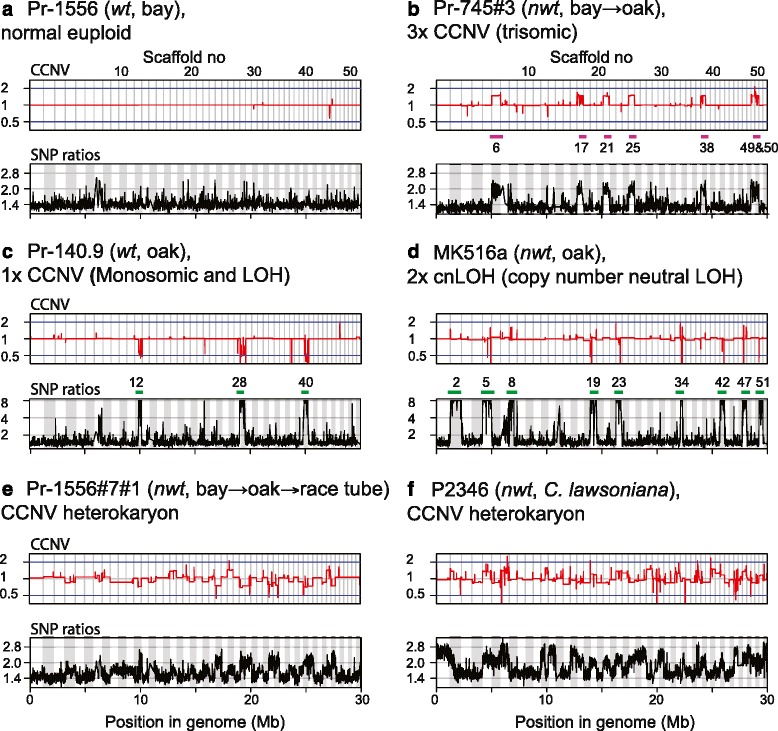
Read-depth analyses revealed chromosomal aberrations in *nwt* isolates. Large CCNVs were revealed by BIC-seq analysis (*upper graph for each panel*) and by a read-depth analysis for heterozygous allele ratios using 10 KB long non-overlapping sliding window (*lower graph*). A concatenated view of the 52 largest scaffolds with a total length of 300 MB, corresponding to approximately half of the total genome of *P. ramorum* is shown. Scaffold numbers for large CCNV regions are indicated with pink bars, and those for LOH are shown with green bars. Scales show log (base 2) fold difference between sample isolates and reference isolates for BIC-seq analysis and log (base 2) ratios of alleles of sample isolates for the heterozygous allele frequency analysis. At each heterozygous locus, a read count ratio (more-abundant allele/less-abundant allele) was calculated. Allele count ratios larger than eight were set to eight to visualize the loss of heterozygosity. For the re-isolates Pr745#3 and Pr1156#7#1, corresponding progenitor isolates were used as reference for BIC-seq analyses (reference isolates listed in Table 3). **a** Pr-1556, an example of CCNV profile for the category “normal euploid”. **b** *Nwt* re-isolate Pr-745#3 from oak, an example of 3x CCNV, showing trisomy (1.5-fold increase in copy number) in the seven scaffolds. **c** *Wt* oak isolate Pr-140.9 (1x CCNV type) showing monosomy (0.5-fold decrease in copy number) in three scaffolds. **d** *Nwt* oak isolate MK516a, an example of copy number neutral LOH (2x cnLOH). Close inspection of short segments with CCNV seen as spikes in cnLOH regions (above the green bars) reveal the *wt* reference genome used for the BIC-seq analysis has heterozygous indels (>100 bp) in these regions. Loss of chromosomal segments harboring these indels in the *nwt* isolate MK516a resulted in spikes in the BIC-seq analysis. **e** A re-isolate from the race tube showed a mixture of nuclei with heterogeneous CCNVs. **f** A *nwt* EU1 isolate P2346 revealed extensive CCNVs and LOH when *wt* EU1 isolate P2363 was used as a reference

The majority (84 %) of CCNVs found among the seventeen *wt* isolates clustered in one of 20 genomic locations, and among these 20 locations, 12 locations were at the vicinity of TEs. Apart from these hypervariable short segments displaying CCNVs, no major chromosomal alterations were detected in the *wt* isolates, which resulted in a largely linear red line across the concatenated scaffolds when *wt* isolates were compared (Fig. 4a, upper graph). Additionally, average heterozygous allele ratios, using 10 KB long non-overlapping sliding window, were close to one (Fig. 4a, lower graph). These observations indicate that the seventeen *wt* isolates have a balanced set of chromosomes. This group of isolates was thus classified as the “normal euploid group” (Fig. 4a and Table 3). In contrast, all seven *nwt* isolates had extensive CCNVs. For instance, when genomes of Pr-745 (*wt*, bay) and one of its re-isolates, Pr-745#3 (*nwt*, bay➔oak) were compared, a 1.5-fold increase in chromosomal content was detected for several scaffolds in Pr-745#3 (Fig. 4b, upper graph). Additionally, average read depths for one of the heterozygous alleles located on duplicated scaffolds were twice those of alternative alleles (Fig. 4b, lower graph). Note that these two independent analyses detected chromosomal alterations at the same locations and that the boundaries of the altered regions coincided completely with those of scaffolds, indicating the precision of the analyses. These observations indicate *de-novo* copy number change from two to three in the oak re-isolate. The sum of the duplicated scaffolds for Pr-745#3 corresponds to nine % of the total genome.

The current assembly of *P. ramorum* is comprised of 2,576 scaffolds [29] and genetic and physical linkages among the scaffolds are currently unknown. Hence, the duplicated scaffolds seen along the concatenated scaffolds are most probably located on the same chromosome. Given that *Phytophthora* species usually have five to 12 major chromosomes [30–32] and *P. ramorum* is believed to be *n* = 10-12 (D. Beattie and C.M. Brasier, unpublished cytological study), we estimate the duplicated regions correspond to trisomy in one or two whole chromosomes.

Two isolates from naturally infected oak, Pr-16 (*nwt*, oak) and Pr-102 (*nwt*, oak), were also found to be trisomic but with a different set of scaffolds (Additional file 3). This group is categorized as “3x CCNV”. In addition, four *nwt* isolates with intermediate values of chromosome copy number (e.g. Fig. 4e and Additional file 3D,E), indicative of mixtures of nuclei with heterogeneous CCNVs [33], were identified. This group is categorized as “CCNV heterokaryon”.

In three oak isolates, large stretches of genomic regions with loss of heterozygosity (LOH) in SNP loci were identified. A *wt* isolate (Pr-140.9, Fig. 4c) was found to have a 0.5-fold decrease in the chromosomal content at the LOH regions. These LOH regions correspond to monosomy, and thus the isolate Pr-140.9 was classified as “1x CCNV”. On the other hand, in the two *nwt* isolates, MK516a (Fig. 4d) and Pr16 (Additional file 3B), chromosomal copy number changes along the stretches of the LOH regions were not detected. These LOH regions correspond to a chromosomal aberration known as uniparental disomy or copy-neutral loss of heterozygosity (cnLOH); these isolates were categorized as “2x cnLOH”.

**Fig. 5:**
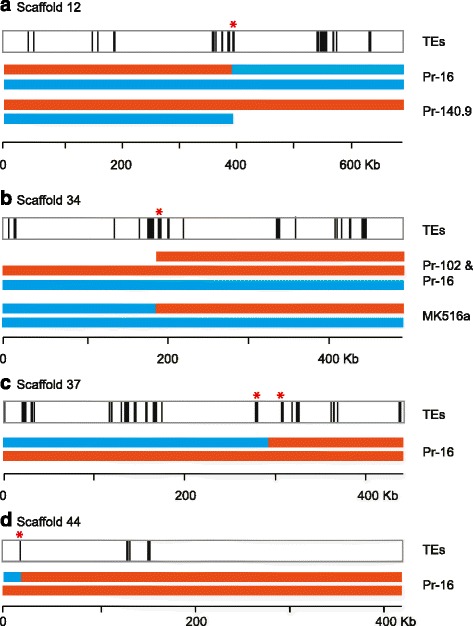
Chromosomal breakpoints were associated with transposable elements. Of the nine NA1 isolates carrying chromosomal aberrations, a total of seven independent chromosomal breakpoints were identified at four chromosomal locations (see Additional files 4, 5, 6 and 7 for details). Black vertical lines represent transposable elements visualized by Integrative Genomics Viewer [81]. Cyan and orange bars represent segments of homologous chromosomes. **a** In scaffold 12, chromosomal breakpoints resulted in a partial monosomy in Pr-140.9 and a cnLOH in Pr-16. Two LINE retrotransposons were located at the breakpoint (*red asterisk*). **b** In scaffold 34, the chromosomal breakpoints for the partial trisomy (Pr-102 and Pr-16) and cnLOH (MK516a) occurred at the same genomic location. Four gypsy retrotransposons were found at the breakpoint (*red asterisk*). **c** Another breakpoint for cnLOH in Pr-16 was found in scaffold 37. Two gypsy retrotransposons flank the breakpoint (*red asterisks*). **d** The third breakpoint for cnLOH in Pr-16 was located in scaffold 44. A MuDR DNA transposon was located at the breakpoint (*red asterisk*). Additional files. 4, 5, 6 and 7 show close up of breakpoints

A cross-examination of CCNV and SNP datasets revealed a total of four chromosomal breakpoints identified as cnLOH transitions in scaffolds 12, 34, 37, and 44. A disomy to partial monosomy transition was also observed in scaffold 12, and a disomy to partial trisomy transition was observed in scaffold 34. All four breakpoints were found in close vicinity to TEs (Fig. 5, Additional files 4, 5, 6 and 7 for details). Note that except for Pr-16, which was categorized as 3x CCNV as well as 2x cnLOH (Table 3 and Additional file 3) and had four breaks identified in its genome, the other three isolates, Pr-140.9, Pr-102, and MK516a had only one break identified per isolate. Transcriptome analysis did not detect transcripts of TEs (scaffolds 12, 34, and 37) nor differential gene activity of TEs between euploid and aneuploid isolates (scaffold 44) at the breakpoints (Additional file 8), implying that activity of TEs and the formation of chromosomal aberrations is a transient phenomenon.

In summary, *nwt* isolates have on average a greater number of larger CCNVs than *wt* isolates do, demonstrating the association of *nwt* and CCNVs. Also, involvement of TEs in the generation of CCNVs was implicated.

### CCNVs in *P. ramorum* EU1 lineage exposed to Lawson cypress

In Europe, two genetically distinct clonal lineages of *P. ramorum* occur, EU1 and EU2 [14, 17]. EU1 isolates, mainly from *Rhododendron* or *Viburnum* spp. and from European Beech (*Fagus sylvatica*), have shown a remarkably high level of stability compared to isolates of the NA1 lineage, and normally exclusively exhibit the *wt* colony morphology [19]. Furthermore >200 EU1 isolates from destructive unprecedented outbreaks on plantation larch (*Larix* spp.) in the UK starting in 2009 [11] have also been described as *wt*. However, in 2010, twenty-five EU1 isolates obtained from the phloem of a single four meter long lesion on a mature Lawson cypress (*Chamaecyparis lawsoniana*) in the UK [34], displayed diverse colony types. It is estimated that from the time of the original infection the pathogen was probably active in this lesion for at least three seasons. Five isolates from the middle of the lesion all had the *wt* phenotype and remained *wt* in re-subculturing (Additional file 9D,E,F). Seven from the top front of the lesion were initially a mixture of *wt* and *nwt* phenotypes, but all became *nwt* on re-subculturing (Additional file 9A,B,C). Many isolation attempts from the bottom lesion front failed, and some isolates senesced before they could be subcultured, but all of the thirteen successful isolates were and remained *nwt* (Additional file 9G,H,I).

Three representative isolates, one reference *wt* from the middle of the lesion and two *nwt* from the top and bottom lesion fronts (Table 3) were subjected to CCNV analysis. Large chromosomal regions with CCNVs and LOH were identified in the EU1 *nwt* isolates in comparison to both the EU1 *wt* (Table 3, Fig. 4f and Additional file 3F) and the NA1 *wt* reference isolates (Additional file 10). Intermediate values of chromosomal copy number and the increase in SNP allele ratios at heterozygous loci indicate the three isolates (one *wt* and two *nwt*) were CCNV heterokaryons.

### CCNVs in relation to the growth acceleration phenotype (GAP)

To obtain information on the development of CCNVs, GAP, and *nwt* phenotype, a CCNV analysis was conducted on the isolates which had been subjected to a series of passage experiments (CCNV generation in Table 3). No major CCNVs or noticeable changes in SNP allele ratios were seen in the inoculum isolate Pr-1556 (*wt*, bay), re-isolate from oak Pr-1556#7 (*wt*, bay➔oak), or the inoculum isolate passed through the race tube, Pr-1556#2 (*wt*, bay➔race tube) (all similar to Fig. 4a). In contrast to the genome of Pr-1556 (*wt*, bay), which was stable over 18 weeks of growth in the race tube (Pr-1556#2), Pr-1556#7#1 (*nwt*, bay➔oak➔race tube) was found to be a CCNV heterokaryon (Fig. 4e), supporting the notion that oak but not California bay destabilizes the genome of *P. ramorum*.

### Genes in duplicated chromosomal regions are over-expressed

Differential expression of genes located on the duplicated regions is expected and may be responsible for the phenotypic changes in oak isolates. Global mRNA profiles of trisomic isolates Pr-102 (*nwt*, oak) and Pr-745#3 (*nwt*, bay➔oak) were compared with those of the four *wt* normal euploid isolates (Fig. 2). As a result, 1,400 and 715 differentially expressed genes in Pr-102 (*nwt*, oak) and Pr-745#3 (*nwt*, bay➔oak), respectively, were identified (Additional file 8). Microarray analysis detected expression of 217 and 913 genes located in duplicated regions in the first 30 Mb of the concatenated scaffolds in Pr-102 and Pr-745#3, respectively. Genes on the duplicated regions were found more likely to be up-regulated (*p* = 3.2 × 10^−5^ for Pr-102 and 2.0 × 10^−5^ for Pr-745#3) but not down-regulated in relation to the genome average. Such overexpression of duplicated genes may be responsible for the observed phenotypic changes.

## Discussion

Epidemiological and experimental studies support the hypothesis that oak is a dead-end for *Phytophthora ramorum* and that propagules produced on infectious hosts such as California bay initiate infection on oak [22, 23]. The fact that unusual non wild-type phenotypic variation (*nwt*) has been observed in oak isolates and not in California bay populations of *P. ramorum* [21] together with the evidence that California bay populations provide the inoculum source for the oak populations suggests that: 1)- the genomic changes involved in *nwt* occur once the pathogen colonizes oak and that 2)- colonization of oak does not exert a similar selection pressure on the *P. ramorum* genome as colonization of California bay.

Since the oak-*nwt* variation apparently occurs in the absence of any genetic differentiation in the *P. ramorum* population [21]*,* the concept of host-induced phenotypic diversification (HIPD) was formulated. The main objective of the present study was to demonstrate HIPD experimentally and gain insight into the underlying genetic mechanisms. When wild type (*wt*) isolates from California bay were artificially inoculated on both oak and California bay, HIPD was observed only when the pathogen was re-isolated from oak. In these tests, colony morphologies and mRNA profiles of wild type (*wt*) isolates converted to non-wild type (*nwt*) through artificial oak inoculation matched the colony morphologies and the mRNA profiles of *nwt* isolates obtained from naturally infected oak. This is further evidence that *nwt* isolates are not a permanent subpopulation specialized in oak but they arise *de novo* inside oak from the general population. The *nwt* colony phenotype was not always observable among re-isolates immediately after retrieval from inoculated hosts, but did develop in re-isolates from oak during subsequent in vitro colony growth.

In the sequence analyses, *nwt* colony morphology was found to be strongly correlated with a range of chromosomal aberrations including aneuploidy and chromosomal number neutral loss of heterozygosity (cnLOH). A similar association of *nwt* phenotype and chromosomal aberrations was observed in the EU1 isolates from Lawson cypress. Induction of aneuploidy has been reported in several fungal pathogens in response to antifungal chemical treatments [6, 9]. Also, some viral, bacterial, and fungal pathogens are known to modify host cell ploidy (e.g. [35–37]). In oomycetous pathogens, field isolates with aneuploidy have been identified in diverse species [38–41] and meiotic generation of aneuploids has also been reported [32, 42, 43]. Likewise, mitotic cnLOH has been detected in field isolates and intercross progeny and shown a strong association with changes in pathogenicity [44]. Our observation of a presumed mitotic generation of aneuploidy and cnLOH in oak and Lawson cypress is to our knowledge the first report of aneuploidy in a defined host-pathogen interaction in nature.

The location of chromosomal breakpoints associated with the genome aberrations may provide clues to the mechanisms underlying the induced aneuploidy and LOH. Of the total of four identified breakpoints in NA1, two (scaffolds 12 and 34 in Fig. 5) were shared among multiple isolates displaying cnLOH and aneuploidy. Given that oak is a dead-end host for *P. ramorum*, with no oak-to-oak infection, the shared chromosome breakpoints must be of independent origin. Evidence from other organisms indicates that formation of breakpoints is non-random and that common genetic mechanisms occur. These include breakage of chromosomes at common fragile sites under replication stress, followed by repair of the double-strand breaks via homologous recombination, resulting in partial aneuploids and cnLOH [45–47]. All four breakpoints in *P. ramorum* NA1 were located at or near TEs. This is in agreement with the observation that TEs were located at the breakpoints of segmental aneuploids in *Saccharomyces cerevisiae* [48]. Association of TEs and chromosomal breakage during mitotic divisions is also well established [49, 50]. For example, Ty retrotransposons have also been found near double-stranded DNA breaks in mitotically dividing cells in *S. cerevisiae* [51]. This is consistent with our finding of retrotransposons near the breakpoints in *P. ramorum* scaffolds 12, 34, and 37.

Three NA1 and three EU1 isolates were shown to be a mixture of euploid and aneuploid nuclei. The spatial distribution of the nuclear mixture could be structured or homogeneous (equally distributed throughout the mycelia). For example, if structured, some hyphae would have exclusively euploid nuclei, and other hyphae exclusively aneuploidy nuclei. If distribution is homogenous, all hyphae would have both nuclear types (i.e. be heterokaryotic). Because (1) oomycete cells are multinucleate and heterogeneous nuclei can coexist within a single cell [52, 53], and because (2) euploid and aneuploid hyphae can have a significant difference in growth rates [54, 55], the cultures with mixed nuclei are most likely to be heterokaryotic.

It is noteworthy that the three EU1 isolates from a single lesion on Lawson cypress showed different ratios of euploid to aneuploid nuclei (Additional file 10). The *wt* isolate P2363 showed the least deviation from one in the ratios of heterozygous SNP alleles when compared to *nwt* isolates P2346 and P2386. Therefore, P2363 hyphae contained the least number of aneuploid nuclei. Hence, it could be that a higher ratio of aneuploid to euploid nuclei in the hyphae results in the *nwt* phenotype. Indeed, such an increase in nuclear ratio could also explain the phenomenon of latent development of *nwt* colony morphology in *wt* isolates during growth in vitro, which has been observed in both the oak and Lawson cypress isolates.

Some recent evidence suggests that the generation of aneuploids and LOH can result in episodic selection (rapid adaptation to environmental shifts) [9, 33]. For example, in the human pathogens *Candida albicans* and *Cryptococcus neoformans,* antifungal drug resistance is conferred by duplication of chromosomes harboring genes for drug targets (reviewed in [9]). A rapid generation of LOH in the presence of an antifungal drug has also been observed for *S. cerevisiae* [56]. It is posited that aneuploidy generates changes in gene dosage and, therefore, creates a phenotypic variation on which selection can act [57]. LOH can remove dominant alleles so that potentially beneficial recessive alleles can contribute to an organism’s phenotype. LOH can, therefore, allow beneficial recessive alleles to escape Haldane’s sieve [56]. Present evidence indicates that in *P. ramorum nwt* individuals are ecologically less fit than *wt* individuals in that they are less pathogenic and slower growing and prone to senescence in culture [19–21]. There could, however, be circumstances where phenotypes driven by host-induced aneuploidy confer survival advantages to pathogens. Thus, we cannot altogether discount the possibility that the resulting phenotypic diversification may allow an introduced pathogen such as *P. ramorum* to adapt to a novel host or to other episodic selection conditions [2, 58].

Although induced chromosomal aberration as a result of host defense is currently unknown to science, the observed *nwt* phenotype may be due to damage inflicted directly by the host’s defense mechanisms, such as induced or constitutive metabolites, resulting in induced chromosomal aberration (see below). Thus the colony instability, slower growth rates and lower aggressiveness of the *nwt* phenotype can be viewed as degenerate and at a fitness disadvantage compared to the *wt* form. Indeed, individual oak trees infected with *P. ramorum* do not always die suddenly. Some live for many years and in others the infection may die out [59]. Such oak survival may be associated with the conversion of *P. ramorum* to slow growing *nwt*, allowing the host to contain it, or the pathogen may become senescent and therefore effectively eradicated, as appears to have occurred at the perimeter of the large lesion on Lawson cypress.

The host factors that trigger HIPD remain unknown. Chronic exposure to specific chemicals in oak and Lawson cypress bark may make nuclei prone to chromosomal abnormalities, whereas exposure to the chemical environment of other hosts such as California bay or *Rhododendron* may not. The phloem of coast live oak contains a mixture of chemicals including phenolics, some of which are associated with field resistance to *P. ramorum* [60, 61]. Likewise, bark and wood of Lawson cypress also contains a wide range of secondary metabolites including polyphenols and oils such as limonene that exhibit antifungal activity [62]*.* Additionally*,* Lawson cypress oil is reported to contain about 2 % camphor [63]. Camphor has been used to induce polyploidy in the true fungi and oomycetes including *Phytophthora* [64]. The 3–4 years that *P. ramorum* spent in the bark of the Lawson cypress canker would have provided ample time for bark compounds to induce chromosomal abnormalities. The possible role of camphor, and other bark constituents of Lawson cypress and oak in inducing *nwt* phenotype in *P. ramorum* could be tested experimentally in further race tube tests.

We have shown that high-throughput sequencing methods for CCNV and LOH detection are fast, reliable, and produce high-resolution data. They should, therefore, be valuable additional tools for understanding the genetics of evolutionary processes not just in *Phytophthora*, but in other organisms as well. The quality of genome assembly is vital to the CCNV and LOH analyses. In future we suggest the resolution and sensitivity of studies on structural variations of chromosomes may be significantly improved by applying long-read sequencing and genome assembly technologies to the assembly of full-length chromosomes [65, 66].

## Conclusions

The Sudden Oak Death pathogen *Phytophthora ramorum* is exclusively clonal, yet exhibits extensive phenotypic differences when obtained from oak. When *P. ramorum* isolates from the foliar host California bay were inoculated and re-isolated from canyon live oak, a large number of re-isolates displayed morphological phenotypes and mRNA expression profiles only seen in cultures from naturally infected oak. Major genomic alterations in oak isolates including partial aneuploidy and copy-neutral loss of heterozygosity were found to be associated with the observed phenotypic diversification. Comparable phenotypic changes and associated genome alterations were also found in isolates from Lawson cypress in the UK. Chromosomal breakpoints were found to be located at or near transposons, linking transposon de-repression caused by the chemical environment of oak to structural genomic changes.

## Methods

In this research, *P. ramorum* isolates derived from California bay were 1) inoculated into oak and California bay, and the pathogen was re-isolated and changes in colony phenotypes were recorded. Microarray mRNA profiling and illumina DNA-seq were used to address processes underlying the phenotypic conversion. All permits to work with *P. ramorum* in the lab and the field in California have been secured from the California Department of Agriculture (Permit No. 2201). Collecting permits have also been obtained for all sites where field plots have been established. These include California State Parks (blanket permit for all state parks), National Park Service (Redwood National Park and associated North Coast State Parks, Pt. Reyes National Seashore), Marin Municipal Water District, Monterey Regional Parks District, East Bay Regional Parks, Big Sur Land Trust, and Midpeninsula Regional Open Space District.

### Isolates and culture conditions

A total of 41 Californian and three British *P. ramorum* isolates were examined in this study ( Additional file 2 for details). Cultures were maintained on small plugs of 6.6 % clarified V8 Juice with 1.5 % agar (1/3 x CV8A) [67] submerged in water at 14 °C.

### Inoculation and re-isolation

Inoculation experiments on oak were conducted twice. In July 2010, we initiated the first experiment by inoculating 18 canyon live oaks and two Shreve oaks at a site in San Mateo County (details in [26]). Holes five mm in diameter were made to the cambial zone of mature trees (25 cm average diameter at breast height) with a cork borer and inoculation was made with a five mm diameter agar plug cut from the margin of a 7-day old culture growing on 1/3 V8A (6.6 % non-clarified V8 with 1.5 % agar), following procedures previously used for coast live oak [10]. Each tree was inoculated with two different local *P. ramorum* isolates, Pr-710 and Pr-745, and a control mock inoculation (sterile agar only). Pr-710 was originally derived from California bay, whereas Pr-745 was derived from rainwater near infected California bay trees. Twenty and 40 weeks after inoculation, cankers that developed under tree bark were exposed and small pieces of phloem tissue cut from canker margins were placed onto PARP selective medium [68] for re-isolation of *P. ramorum*. The second oak inoculation experiment was initiated in July 2012. Four canyon live oak trees (previously inoculated, but appeared healthy) were selected and two other local *P. ramorum* isolates, Pr-1556 and Pr-1557, both derived from California bay, were inoculated to locations distant enough to avoid merging into the earlier July 2010 inoculations. Re-isolation was performed 20 weeks after inoculation.

Inoculation experiments on California bay were also conducted twice. In April 2012, 10 leaves of one 18-L potted California bay seedling were wounded with a surgical scalpel. Inoculum plugs five mm in diameter were taken from an actively growing margin of *P. ramorum* colony of isolate Pr-745 (*wt*, bay) on 1/3x V8A. Plugs were placed mycelium side up into a screw cap lid from a microcentrifuge tube filled with sterilized dH_2_O and then attached to the underside of the leaves with a pin curl clips. A clear plastic bag filled with 10 ml of sterilized dH_2_O was placed over each inoculated leaf. Bags and clips were removed three days post-inoculation. Plants were kept in 18 °C growth chamber and re-isolations were made with PARP selective medium 20 weeks post-inoculation. The second California bay inoculation experiment was initiated in January 2013. Fifteen leaves each of six 18-L potted California bay seedlings were inoculated with isolates ND886 (*wt*, camellia) and Pr-102 (*nwt*, oak), three seedlings each. Plants were kept outdoors at the National Ornamentals Research Sites at Dominican University of California (NORS-DUC), and re-isolations were made 4, 12 and 25 weeks post-inoculation from three leaves per plant at each time point.

### Evaluation of colony morphology

*P. ramorum* isolates were first grown on 1/3x CV8A at 21 °C for seven days and then subcultured to 1x CV8A (20 % clarified V8 Juice with 1.5 % agar) and grown for another seven days. Colony diameters were measured and colony patterns were photo-documented for at least two replicates per isolate. Colonies that had a uniform circular growth pattern were scored as “wild type” (*wt*). Criteria for *nwt* morphology are as follows. If (1) the growth rate was slower than *wt* by at least 25 % of the average linear growth rate of *wt* or (2) at least 15 % deviation of radius was observed within a 45° sector in a single colony, these colonies were scored as “non-wild type” (*nwt*). When *nwt* colony morphology was observed upon further subculturing, the phenotype of the isolate was described as *nwt* even though the original isolate did not consistently display *nwt* colony morphology. When growth arrest was observed, isolates were scored as displaying “early senescence phenotype”.

### Race tube growth experiment

A race tube is a 40 cm long glass tube that is bent up at both ends to hold agar medium (purchased from Fungal Genetic Stock Center, Kansas City, Missouri USA) [69]. 20 ml of autoclaved 1x CV8A (20 min. 121 °C) was poured into the autoclaved race tube, then both ends were capped with plastic lids. After the medium had solidified, a small piece of mycelium was inoculated at one end of the race tube, Parafilm was wrapped around the lids and incubated at 21 °C under constant cool white fluorescent light at 3.4 μmol m^−2^ s^−1^. The mycelial front was marked once a week to measure growth rate. When the mycelium reached the other end of the race tube, which was after 18 to 25 weeks, the isolate was recovered, grown on 1x CV8A and colony phenotypes were recorded. Owing to the genetic instability of archival oak isolates [21], this experiment could not be repeated for some isolates.

### Growth conditions and RNA extraction for microarray analysis

Growth conditions and RNA extraction [21, 70] were described previously. Mycelia grown on Petri plates (60 mm diameter, catalog no. 351007; Corning Inc.) containing seven ml of 1x CV8A overlaid with a polycarbonate membrane filter (catalog no. 28157–927; VWR) for seven days at 21 °C under constant cool white fluorescent light were subjected to RNA extraction. FastPrep-24 automated cell disruptor was used to homogenize tissues followed by the TRIzol RNA extraction (Invitrogen Life Technologies). Up to 100 μg of total RNA was further cleaned using the RNeasy mini protocol for RNA cleanup (Qiagen). cDNA synthesis, labeling, hybridization procedure, data acquisition and normalization were carried out for NimbleGen microarray analysis according to the manufacturer’s instructions (Roche NimbleGen). Global expression patterns of eight *P. ramorum* isolates were investigated where no biological or technical replicates were included. Results were subsequently validated by qRT-PCR using independently generated RNA samples as described in [21]. Quantile normalization and background correction across arrays were performed using Robust Multi-chip Average (RMA) algorithm [71] implemented in the NimbleScan Version 2.5 software. A MIAME-compliant microarray dataset [72] has been deposited in NCBI GEO database (accession number GSE62643). Additional file 8 lists normalized mRNA profiling results and functional annotations.

### Microarray data analysis

Two isolates derived from California bay, one from coast live oak and five re-isolates from canyon live oak were subjected to microarray analysis. Microarray design and data analysis can be found in our previous report [21]. An additional 2,506 cDNA sequences, which were not represented in the 15,743 gene models predicted in *Phytophthora ramorum* v1.1 (http://genome.jgi-psf.org/Phyra1_1/Phyra1_1.home.html), were also included in the array. Normalized intensity data for 18,001 genes across eight arrays were obtained by RMA and genes with average hybridization intensity below 64 were removed from the dataset. The remaining 14,339 genes were used for further analysis. Pearson’s correlation coefficient between global mRNA expression patterns was then used to cluster cDNA samples using the hclust function with the single linkage option in the statistical software R 3.1.0.

### DNA extraction for Illumina DNA sequencing

A small mycelial plug was transferred to each Petri plate (6 mm diameter, catalog no. 351007; Corning Inc.) containing seven ml of 1x CV8A overlaid with a polycarbonate membrane filter (catalog no. 28157–927; VWR), and grown for seven days at 21 °C in dark. Each circular mycelial mat was cut in half, and lifted from the polycarbonate membrane surface; each half was transferred to a two ml screw-cap microcentrifuge tube (catalog no. 72.694.996 Sarstedt; Fisher Scientific, Santa Clara, CA, USA) and immediately snap-frozen in liquid nitrogen. Each sample weighed approximately 100 mg and was kept at −80 °C until ready for processing. Lysing Matrix A (MP Biomedicals) chilled at −20 °C was added to each of the frozen samples and chilled in liquid nitrogen. Cells were then disrupted once using a FastPrep-24 automated cell disruptor, set at six meters/s for 40 s, in a CoolPrep adapter filled with crushed dry ice. Cells were chilled in liquid nitrogen and disrupted once more for 40 s. 1.5 ml of lysis buffer (20 mM EDTA, 10 mM Tris-Cl, pH7.9, 0.7 mg/ml of enzyme mix, 1 % Triton X-100, 500 mM Guanidine-HCl, and 200 mM NaCl) was added to the pulverized sample, and genomic DNA was extracted according to the User-Developed Protocol for filamentous fungi using the Qiagen Genomic-tip 20/G [73]. A combination of enzymes developed for digesting cell wall material of *Phytophthora infestans* was used [74, 75]; 10x concentration of enzyme mix stock solution comprised of 5 mg/ml Lysing Enzymes from *Trichoderma harzianum* (Sigma-Aldrich) and 2 mg/ml cellulose Onozuka R-10 (Research Products International Corp) was prepared and stored at −20 °C until use, and an appropriate volume was added to prepare a final 1x concentration in the lysing buffer. This method yielded up to 10 μg of genomic DNA. Paired-end libraries were constructed according to the manufacturer’s instructions for TruSeq DNA HT Sample Prep Kit (Illumina, Inc). Conditions and output of Illumina sequencing are in Additional file 11.

### DNA-seq data analysis

A Bayesian adapter trimmer program, Scythe [76] and quality based trimmer, Sickle [77] were used to clean Illumina reads. The processed reads were aligned to the reference genome of *P. ramorum* isolate Pr-102 [29] using Burrow-Wheeler Aligner (BWA) [78] with default parameters. SAMtools was used to process aligned reads [79] and single nucleotide polymorphisms (SNPs) were identified from the aligned reads using Bcftools [80]. Any SNPs whose phred-scaled quality score was 20 or below were filtered out. Chromosomal copy number variation (CCNV) was evaluated by two methods. The first method detects CCNVs from BWA aligned reads using a read-depth algorithm called BIC-seq [28]. To minimize experimental noise, one reference California bay isolate was chosen for each sequencing run and used to estimate CCNVs in the samples processed and run on the Illumina genome sequencer at the same time (Table 3). Because CCNVs between California bay isolates were small, different California bay isolates effectively served as references. The current assembly of the *P. ramorum* genome is comprised of 2,576 scaffolds. These scaffolds were concatenated in order from largest to smallest and CCNVs identified. The second method infers CCNV from read-depth ratios of alleles at heterozygous sites. In the Bcftools output, any heterozygous loci with phred quality scores smaller than 30 were filtered out. Also, any loci carrying alleles with strand bias deviating from a 1:1 ratio (chi-square test *p* < 0.01) were filtered out. Any loci with read ratios of heterozygous alleles equal or larger than eight were also excluded. An average ratio of reads of heterozygous alleles in sliding non-overlapping windows of 10 Kb across each scaffold was then used to infer CCNVs [6]. Loss of heterozygosity (LOH) was also inferred from read-depth ratios of alleles at heterozygous sites. This method is identical to the second CCNV analysis mentioned above but strand bias was not considered, and any allele ratios equal or larger than eight were set to 8. Re-sequencing of 37 isolates identified on average a SNP in every 320 bp in the genome of *P. ramorum*. These SNPs and CCNVs were used to locate chromosomal breakpoints manually using the Integrative Genomics Viewer [81]. Genome coordinates of transposable elements, which can be found at Eumicrobedb.org [82], were kindly provided by Dr. Rays Jiang, University of South Florida.

### Data availability

Additional file 8 contains genome information and mRNA profiling results. The accession number for a MIAME-compliant microarray dataset deposited in NCBI GEO database is GSE62643. BAM alignment files for illumina DNA sequence data were deposited in the NCBI Sequence Read Archive under BioProject accession number SRP061242.

## Additional files

Additional file 1:
*Nwt* colony morphology seen among Petri plate replicates. *Nwt* is observed in **A)** oak isolate Pr-102 (*nwt* colonies indicated with asterisks) but has not been observed for **B)** Pr-745 from rainwater collected near infected California bay. Colonies were grown on solid 1x CV8A medium for 7 days at 21°C in dark. Criteria for *nwt* morphology can be found in Evaluation of colony morphology in Methods. (PDF 18884 kb) Additional file 2: Details of *P. ramorum* isolates used in this study. Other names, source, geographical location, year of isolation and contact scientists for isolates are shown. (XLSX 48 kb) Additional file 3: Diverse CCNVs revealed by BIC-seq analysis (upper graph for each panel) and a read-depth analysis for heterozygous allele ratios using 10 Kb long non-overlapping sliding window (lower graph). A concatenated view of the 52 largest scaffolds with the total length of 300 MB, which corresponding to approximately a half of the total genome of *Phytophthora ramorum*, are shown. Scaffolds numbers for large CCNV regions are indicated with pink bars, and those for LOH are shown with green bars. Scales show log (base 2) fold difference between sample isolates and reference isolates for BIC-seq analysis and log (base 2) ratios of alleles of sample isolates for the heterozygous allele ratio analysis. At each heterozygous locus, a read count ratio (more-abundant allele/less-abundant allele) was calculated. **A)** Pr-102, the genome sequence isolate is trisomic. **B)** Oak isolate Pr-16 is trisomic as well as cnLOH. **C)** Trisomy persists in a re-isolate of Pr-102 from California bay. **D)** A re-isolate Pr745#4 and **E)** an oak isolate Pr-140.7 are CCNV heterokaryons. **F)** *nwt* EU1 isolate P2386 revealed CCNVs and LOH when *wt* EU1 isolate P2363 was used as a reference. (PDF 1014 kb) Additional file 4: The chromosomal breakpoint in scaffold 12. From the right most heterozygous SNP and the left most SNP with LOH, the breakpoints for Pr-140.9 (monosomy) and Pr-16 (cnLOH) were inferred to be within the 1.3kb red rectangle region (top panel, Integrative Genomic Viewer ver. 2.3.34). BICseq CCNV analysis for Pr-140.9 inferred the breakpoint in the close proximity to the red rectangle (middle panel). Homologous chromosomes are depicted in orange and cyan (lower panel). (PDF 57 kb) Additional file 5: The chromosomal breakpoint in scaffold 34. From the right end of the 5.0 kb indel and the left most heterozygous SNP, the breakpoint for MK516a (cnLOH) is inferred to be somewhere within the 7.5 kb red rectangle region (top panel, Integrative Genomics Viewer ver. 2.3.34). For Pr-102 (trisomy) and Pr-16 (trisomy), ratios of heterozygous SNP reads are not reliable for inference for the precise transition from disomy to trisomy. BICseq CCNV analysis, however, located the breakpoints for Pr-102 and Pr-16 at positions 191,470 bp and 190,370 bp, respectively (only Pr-16 is shown in the middle panel), which are within the red rectangle range. Homologous chromosomes are depicted in orange and cyan (lower panel). (PDF 63 kb) Additional file 6: The chromosomal breakpoint in scaffold 37. From the right most heterozygous SNP and the left most SNP with LOH, the breakpoint for Pr-16 (cnLOH) was inferred to be within the 3.0kb red rectangle region (top panel, Integrative Genomic Viewer ver. 2.3.34). Note that Pr-1556 is a normal diploid isolate having heterozygous SNP loci on the right of the breakpoint. Homologous chromosomes are depicted in orange and cyan (lower panel). (PDF 51 kb) Additional file 7: The chromosomal breakpoint in scaffold 44. From the right most heterozygous SNP and the left most SNP with LOH, the breakpoint for Pr-16 (cnLOH) was inferred to be within the 1.3kb red rectangle region (top panel, Integrative Genomic Viewer ver. 2.3.34). Homologous chromosomes are depicted in orange and cyan (lower panel). (PDF 46 kb) Additional file 8: Genome information and mRNA profiling results. This dataset provides mRNA profiling results and information for each gene such as functional annotation, GO terms, transposon class and expression clusters for 12,450 detected genes by means of *P. ramorum* NimbleGen expression microarray. Each of columns is explained in the attached worksheet “Readme”. (XLSX 3418 kb) Additional file 9:
*Nwt* colony morphology seen among EU1 isolates obtained from a single 4 m long lesion on a mature Lawson cypress. EU1 isolates **(A-C)** are derived from the top, **(D-F)** are from the middle, and **(G-I)** are from the bottom of the lesion. *Mnwt* and *snwt* indicate moderate and severe non-wild types, respectively. (PDF 234 kb) Additional file 10: BIC-seq analysis for three EU1 isolates using an NA1 isolate Pr-1556 (*wt*, bay) as a reference. Due to the genome divergence between the EU1 and NA1 lineages, a high background was seen. For an explanation of graphs, see Additional file 3. **A)** When the genome contents of EU1 *wt* isolate P2363 and the NA1 reference were compared, copy number reduction was detected for several regions (red bars), which accompany increase in SNP allele ratios, suggesting P2363 is a mixture of monosomic and disomic nuclei. **B)** When EU1 *nwt* isolate P2346 and the reference were compared, increases (red bars) as well as decreases in the genome content were detected. Note that several scaffolds show large heterozygous SNP ratios while copy number changes in corresponding scaffolds are subtle. This may implicate a formation of cnLOH. **C)** EU1 *nwt* isolate P2386 shows large SNP allele ratios in several scaffolds indicating the formation of cnLOH (red bars). These cnLOH scaffolds are, however, distinct from those observed in P2346. (PDF 561 kb) Additional file 11: Illumina DNA sequencing summary. Sequencing conditions, facility names, obtained read numbers and genome depth are shown. (XLSX 43 kb)

## Abbreviations

CCNV: chromosomal copy number variation
cnLOH: chromosomal number neutral loss of heterozygosity
GAP: growth acceleration phenotype
HIPD: host-induced phenotypic diversification
LOH: loss of heterozygosity
*nwt*: non-wild type
TE: transposable element
*wt*: wild type
