# Supplementary material for: Host-induced aneuploidy and phenotypic diversification in the Sudden Oak Death pathogen Phytophthora ramorum
Source: BMC Genomics. 2016 May 20;17:385. doi: 10.1186/s12864-016-2717-z (PMC4875591; doi:10.1186/s12864-016-2717-z)
Supplement: Additional file 1: — Nwt colony morphology seen among Petri plate replicates. Nwt is observed in A) oak isolate Pr-102 (nwt colonies indicated with asterisks) but has not been observed for B) Pr-745 from rainwater collected near infected California bay. Colonies were grown on solid 1x CV8A medium for 7 days at 21°C in dark. Criteria for nwt morphology can be found in Evaluation of colony morphology in Methods. (PDF 18884 kb) [file 12864_2016_2717_MOESM1_ESM.pdf]

A) Pr-102 (*nwt*, oak)

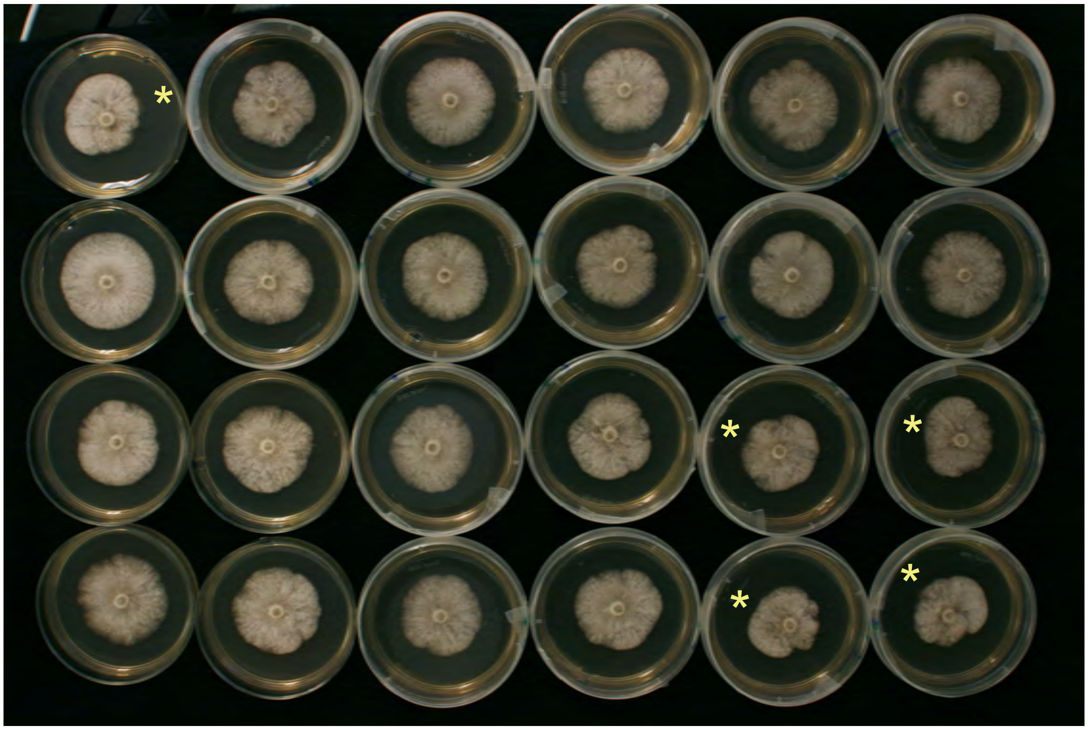

B) Pr-745 (*wt*, bay)

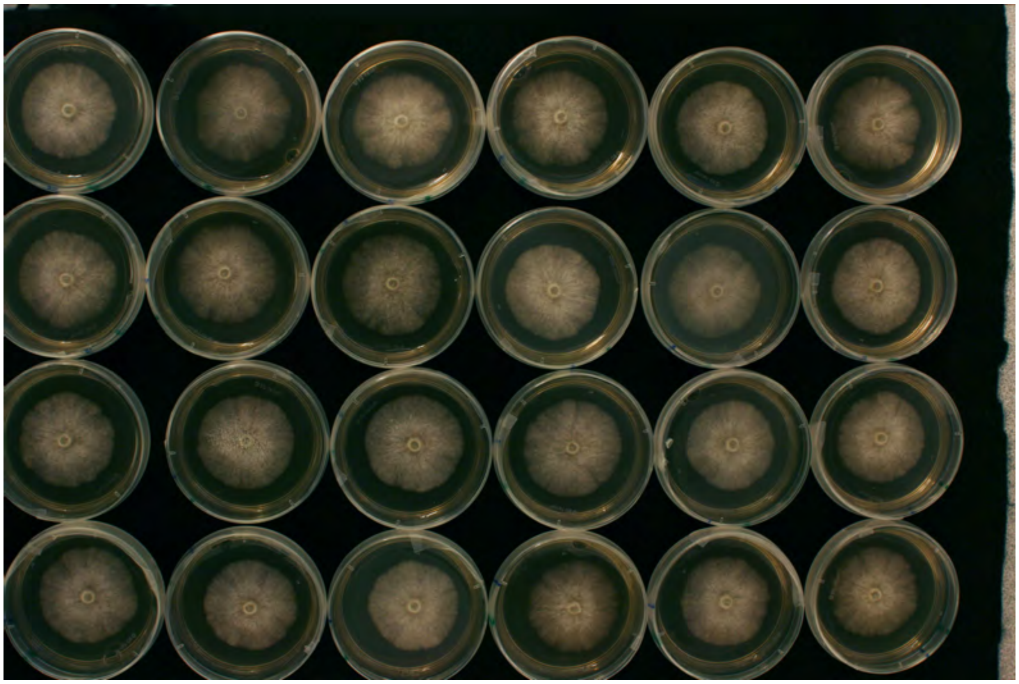

Additional file 1. *Nwt* colony morphology seen among Petri plate replicates. *Nwt* is observed in A) oak isolate Pr-102 (*nwt* colonies indicated with asterisks) but has not been observed for B) Pr-745 from rainwater collected near infected California bay. Colonies were grown on solid 1x CV8A medium for 7 days at 21°C in dark. Criteria for *nwt* morphology can be found in Evaluation of colony morphology in Methods.
