# Supplementary material for: Host-induced aneuploidy and phenotypic diversification in the Sudden Oak Death pathogen Phytophthora ramorum
Source: BMC Genomics. 2016 May 20;17:385. doi: 10.1186/s12864-016-2717-z (PMC4875591; doi:10.1186/s12864-016-2717-z)
Supplement: Additional file 3: — Diverse CCNVs revealed by BIC-seq analysis (upper graph for each panel) and a read-depth analysis for heterozygous allele ratios using 10 Kb long non-overlapping sliding window (lower graph). A concatenated view of the 52 largest scaffolds with the total length of 300 MB, which corresponding to approximately a half of the total genome of Phytophthora ramorum, are shown. Scaffolds numbers for large CCNV regions are indicated with pink bars, and those for LOH are shown with green bars. Scales show log (base 2) fold difference between sample isolates and reference isolates for BIC-seq analysis and log (base 2) ratios of alleles of sample isolates for the heterozygous allele ratio analysis. At each heterozygous locus, a read count ratio (more-abundant allele/less-abundant allele) was calculated. A) Pr-102, the genome sequence isolate is trisomic. B) Oak isolate Pr-16 is trisomic as well as cnLOH. C) Trisomy persists in a re-isolate of Pr-102 from California bay. D) A re-isolate Pr745#4 and E) an oak isolate Pr-140.7 are CCNV heterokaryons. F) nwt EU1 isolate P2386 revealed CCNVs and LOH when wt EU1 isolate P2363 was used as a reference. (PDF 1014 kb) [file 12864_2016_2717_MOESM3_ESM.pdf]

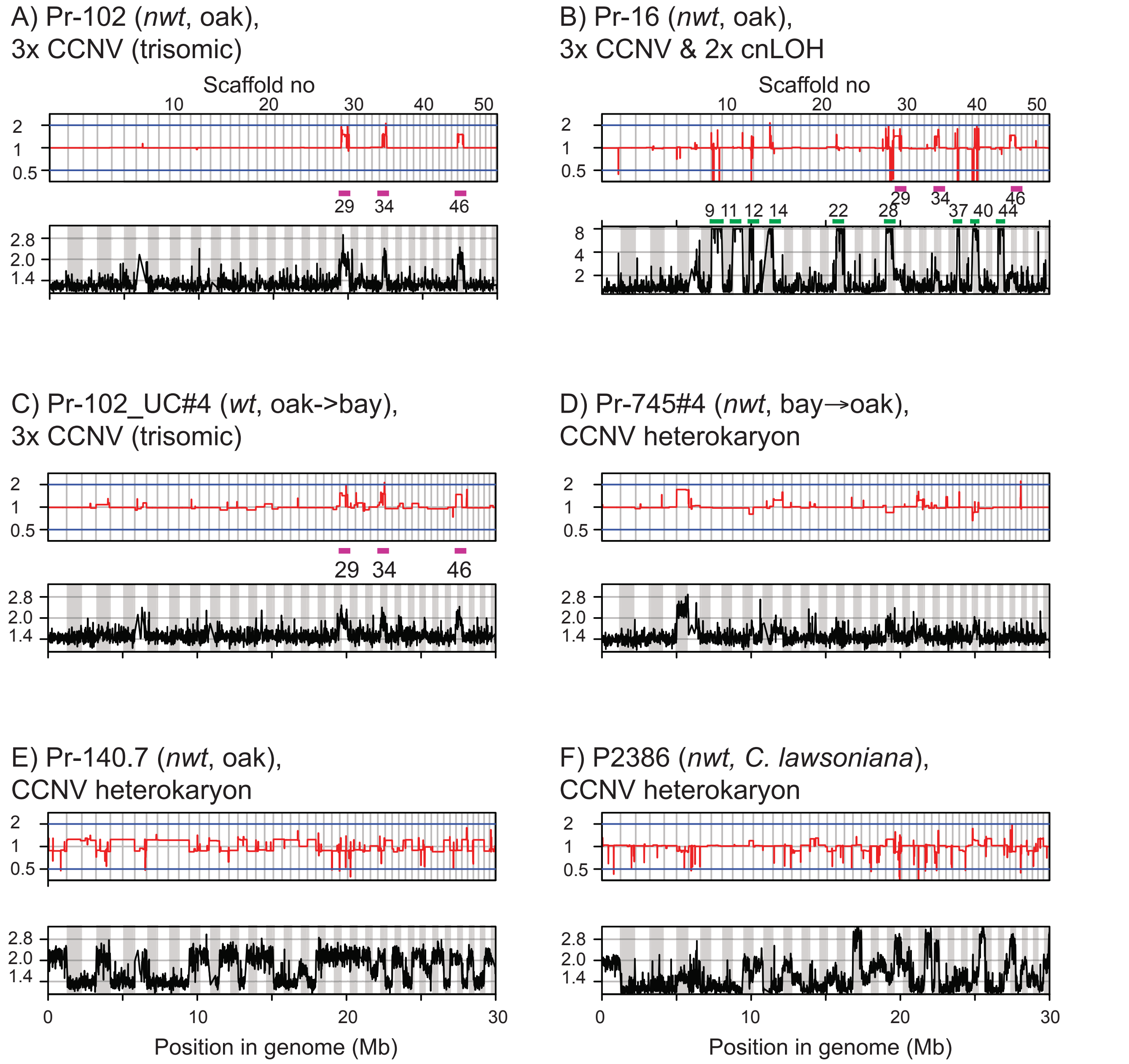

Additional file 3. Diverse CCNVs revealed by BIC-seq analysis (upper graph for each panel) and a read-depth analysis for heterozygous allele ratios using 10 Kb long non-overlapping sliding window (lower graph). A concatenated view of the 52 largest scaffolds with the total length of 300 MB, which corresponding to approximately a half of the total genome of *Phytophthora ramorum* are shown. Scaffolds numbers for large CCNV regions are indicated with pink bars and those for LOH are shown with green bars. Scales show log (base 2) fold difference between sample isolates and reference isolates for BIC-seq analysis and log (base 2) ratios of alleles of sample isolates for the heterozygous allele ratio analysis. At each heterozygous locus, a read count ratio (more-abundant allele/less-abundant allele) was calculated. A) Pr-102, the genome sequence isolate is trisomic. B) Oak isolate Pr-16 is trisomic as well as cnLOH. C) Trisomy persists in a re-isolate of Pr-102 from California bay. D) A re-isolate Pr745#4 and E) an oak isolate Pr-140.7 are CCNV heterokaryons. F) *nwt* EU1 isolate P2386 revealed extensive CCNVs when *wt* EU1 isolate P2363 was used as a refer-
