# Supplementary material for: Host-induced aneuploidy and phenotypic diversification in the Sudden Oak Death pathogen Phytophthora ramorum
Source: BMC Genomics. 2016 May 20;17:385. doi: 10.1186/s12864-016-2717-z (PMC4875591; doi:10.1186/s12864-016-2717-z)
Supplement: Additional file 4: — The chromosomal breakpoint in scaffold 12. From the right most heterozygous SNP and the left most SNP with LOH, the breakpoints for Pr-140.9 (monosomy) and Pr-16 (cnLOH) were inferred to be within the 1.3kb red rectangle region (top panel, Integrative Genomic Viewer ver. 2.3.34). BICseq CCNV analysis for Pr-140.9 inferred the breakpoint in the close proximity to the red rectangle (middle panel). Homologous chromosomes are depicted in orange and cyan (lower panel). (PDF 57 kb) [file 12864_2016_2717_MOESM4_ESM.pdf]

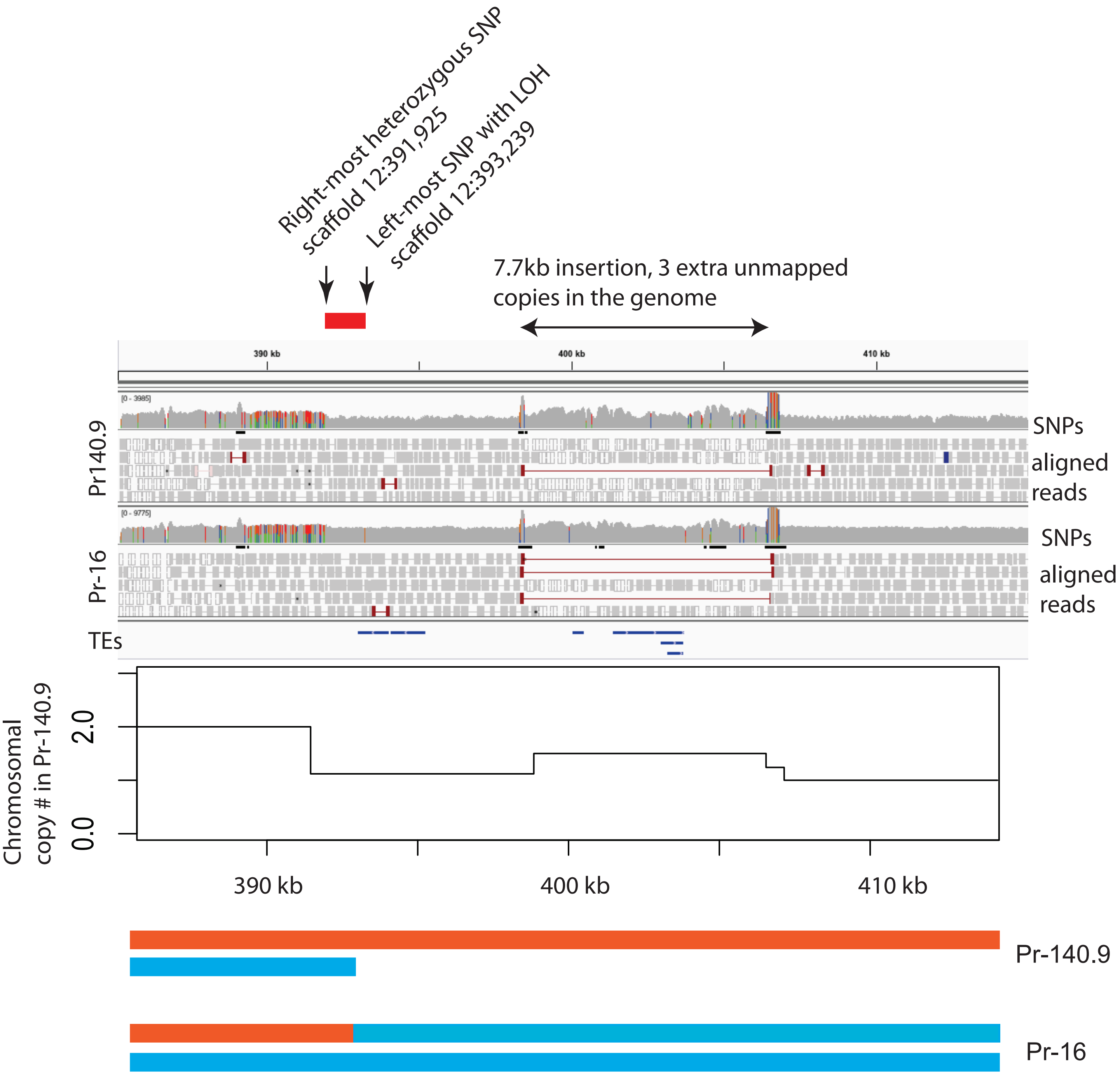

Additional file 4. The chromosomal breakpoint in scaffold 12. From the right most heterozygous SNP and the left most SNP with LOH, the breakpoints for Pr-140.9 (monosomy) and Pr-16 (cnLOH) were inferred to be within the 1.3kb red rectangle region (top panel, Integrative Genomic Viewer ver. 2.3.34). BICseq CCNV analysis for Pr-140.9 inferred the breakpoint in the close proximity to the red rectangle (middle panel). Homologous chromosomes are depicted in orange and cyan (lower panel).
