# Supplementary material for: Host-induced aneuploidy and phenotypic diversification in the Sudden Oak Death pathogen Phytophthora ramorum
Source: BMC Genomics. 2016 May 20;17:385. doi: 10.1186/s12864-016-2717-z (PMC4875591; doi:10.1186/s12864-016-2717-z)
Supplement: Additional file 5: — The chromosomal breakpoint in scaffold 34. From the right end of the 5.0 kb indel and the left most heterozygous SNP, the breakpoint for MK516a (cnLOH) is inferred to be somewhere within the 7.5 kb red rectangle region (top panel, Integrative Genomics Viewer ver. 2.3.34). For Pr-102 (trisomy) and Pr-16 (trisomy), ratios of heterozygous SNP reads are not reliable for inference for the precise transition from disomy to trisomy. BICseq CCNV analysis, however, located the breakpoints for Pr-102 and Pr-16 at positions 191,470 bp and 190,370 bp, respectively (only Pr-16 is shown in the middle panel), which are within the red rectangle range. Homologous chromosomes are depicted in orange and cyan (lower panel). (PDF 63 kb) [file 12864_2016_2717_MOESM5_ESM.pdf]

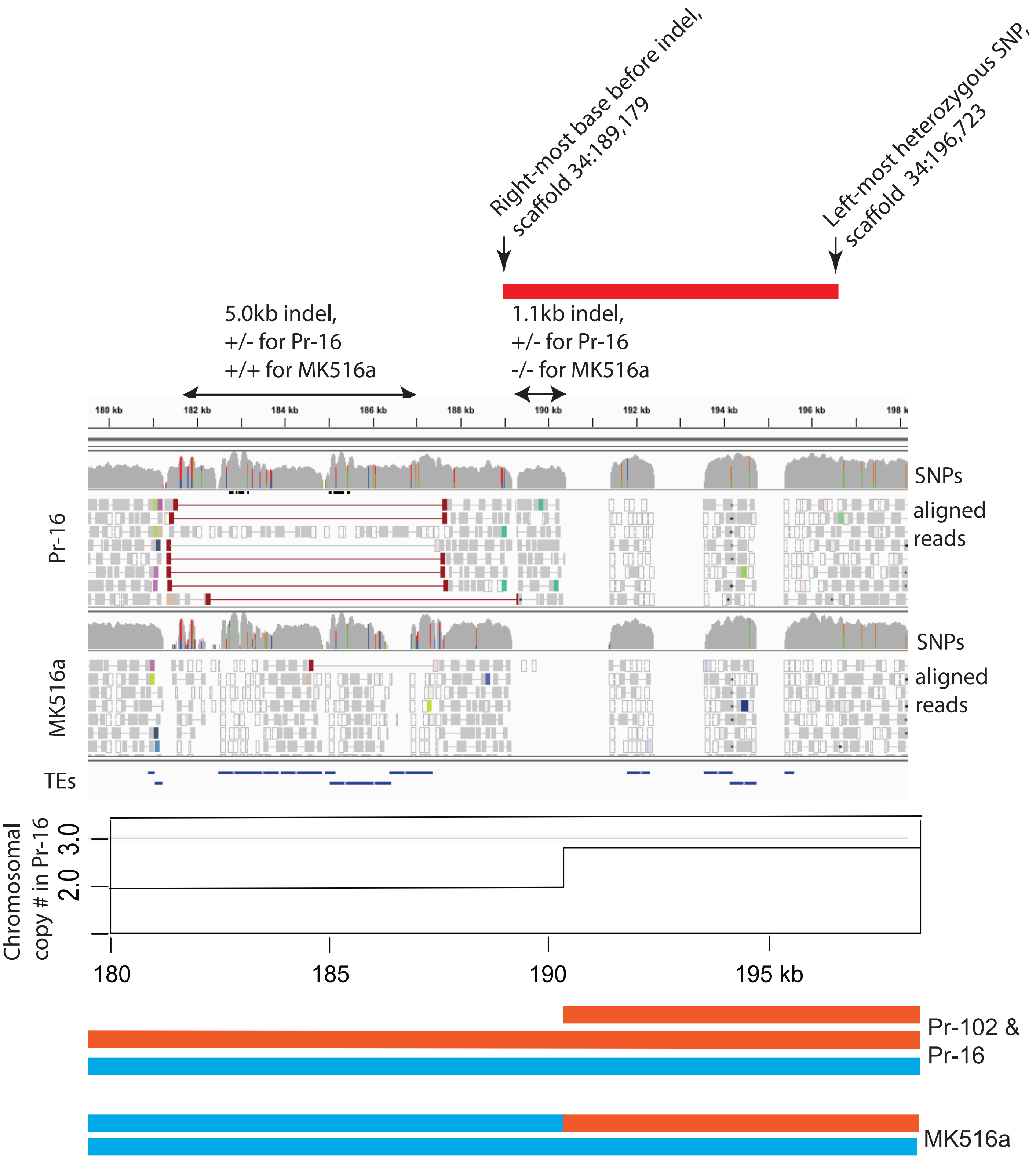

Additional file 5. The chromosomal breakpoint in scaffold 34. From the right end of the 5.0 kb indel and the left most heterozygous SNP, the breakpoint for MK516a (cnLOH) is inferred to be somewhere within the 7.5 kb red rectangle region (top panel, Integrative Genomics Viewer ver. 2.3.34). For Pr-102 (trisomy) and Pr-16 (trisomy), ratios of heterozygous SNP reads are not reliable for inference for precise transition from disomy to trisomy. BICseq CCNV analysis, however, located the breakpoints for Pr-102 and Pr-16 at positions 191,470 bp and 190,370 bp, respectively (middle panel), which are within the red rectangle range. Homologous chromosomes are depicted in orange and cyan (lower panel).
