# Supplementary material for: Host-induced aneuploidy and phenotypic diversification in the Sudden Oak Death pathogen Phytophthora ramorum
Source: BMC Genomics. 2016 May 20;17:385. doi: 10.1186/s12864-016-2717-z (PMC4875591; doi:10.1186/s12864-016-2717-z)
Supplement: Additional file 6: — The chromosomal breakpoint in scaffold 37. From the right most heterozygous SNP and the left most SNP with LOH, the breakpoint for Pr-16 (cnLOH) was inferred to be within the 3.0kb red rectangle region (top panel, Integrative Genomic Viewer ver. 2.3.34). Note that Pr-1556 is a normal diploid isolate having heterozygous SNP loci on the right of the breakpoint. Homologous chromosomes are depicted in orange and cyan (lower panel). (PDF 51 kb) [file 12864_2016_2717_MOESM6_ESM.pdf]

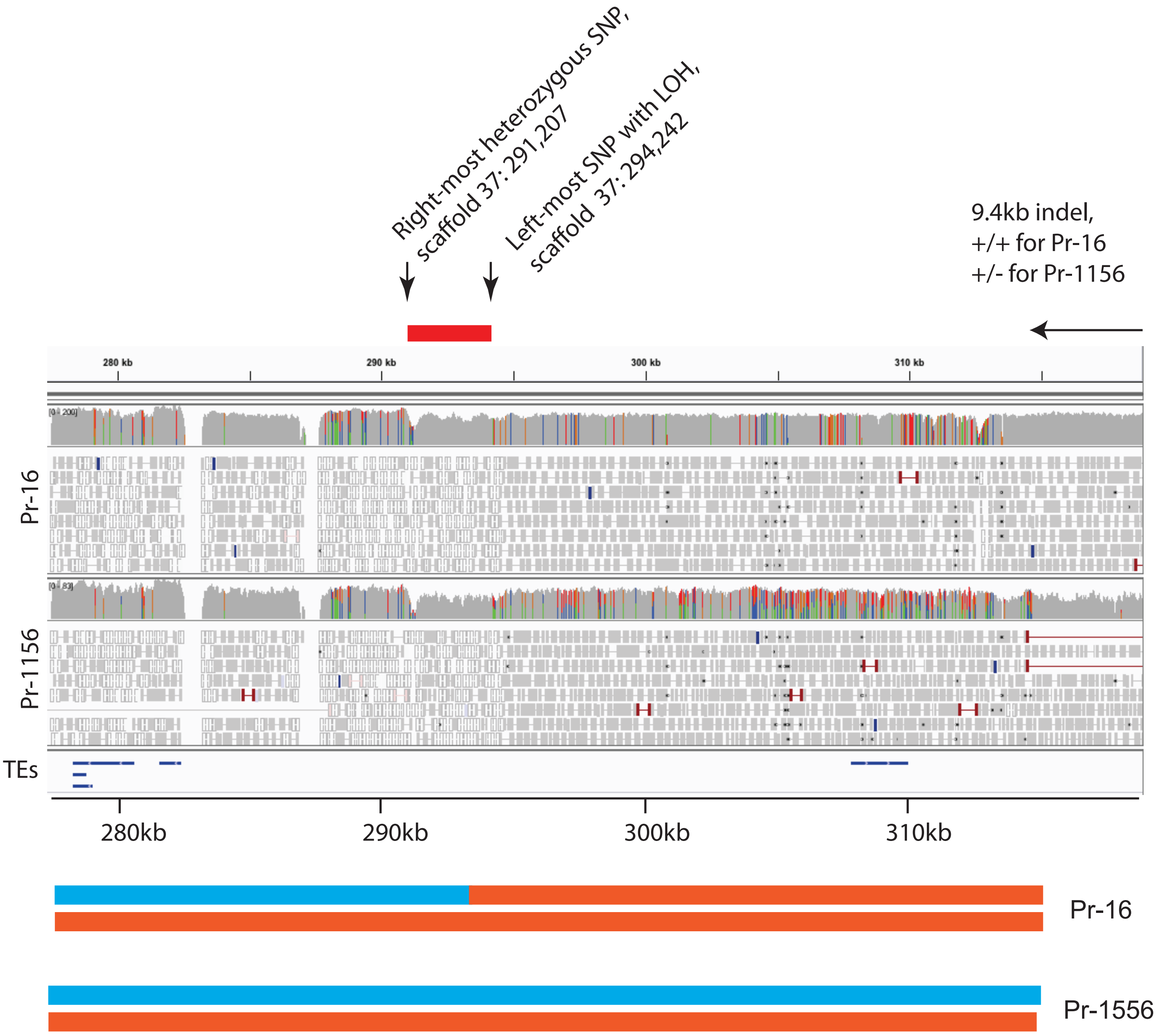


**Additional file 6. The chromosomal breakpoint in scaffold 37.** From the right most heterozygous SNP and the left most SNP with LOH, the breakpoint for Pr-16 (cnLOH) was inferred to be within the 3.0kb red rectangle region (top panel, Integrative Genomic Viewer ver. 2.3.34). Note that Pr-1556 is a normal diploid isolate having heterozygous SNP loci on the right of the breakpoint. Homologous chromosomes are depicted in orange and cyan (lower panel).
