# Supplementary material for: Host-induced aneuploidy and phenotypic diversification in the Sudden Oak Death pathogen Phytophthora ramorum
Source: BMC Genomics. 2016 May 20;17:385. doi: 10.1186/s12864-016-2717-z (PMC4875591; doi:10.1186/s12864-016-2717-z)
Supplement: Additional file 7: — The chromosomal breakpoint in scaffold 44. From the right most heterozygous SNP and the left most SNP with LOH, the breakpoint for Pr-16 (cnLOH) was inferred to be within the 1.3kb red rectangle region (top panel, Integrative Genomic Viewer ver. 2.3.34). Homologous chromosomes are depicted in orange and cyan (lower panel). (PDF 46 kb) [file 12864_2016_2717_MOESM7_ESM.pdf]

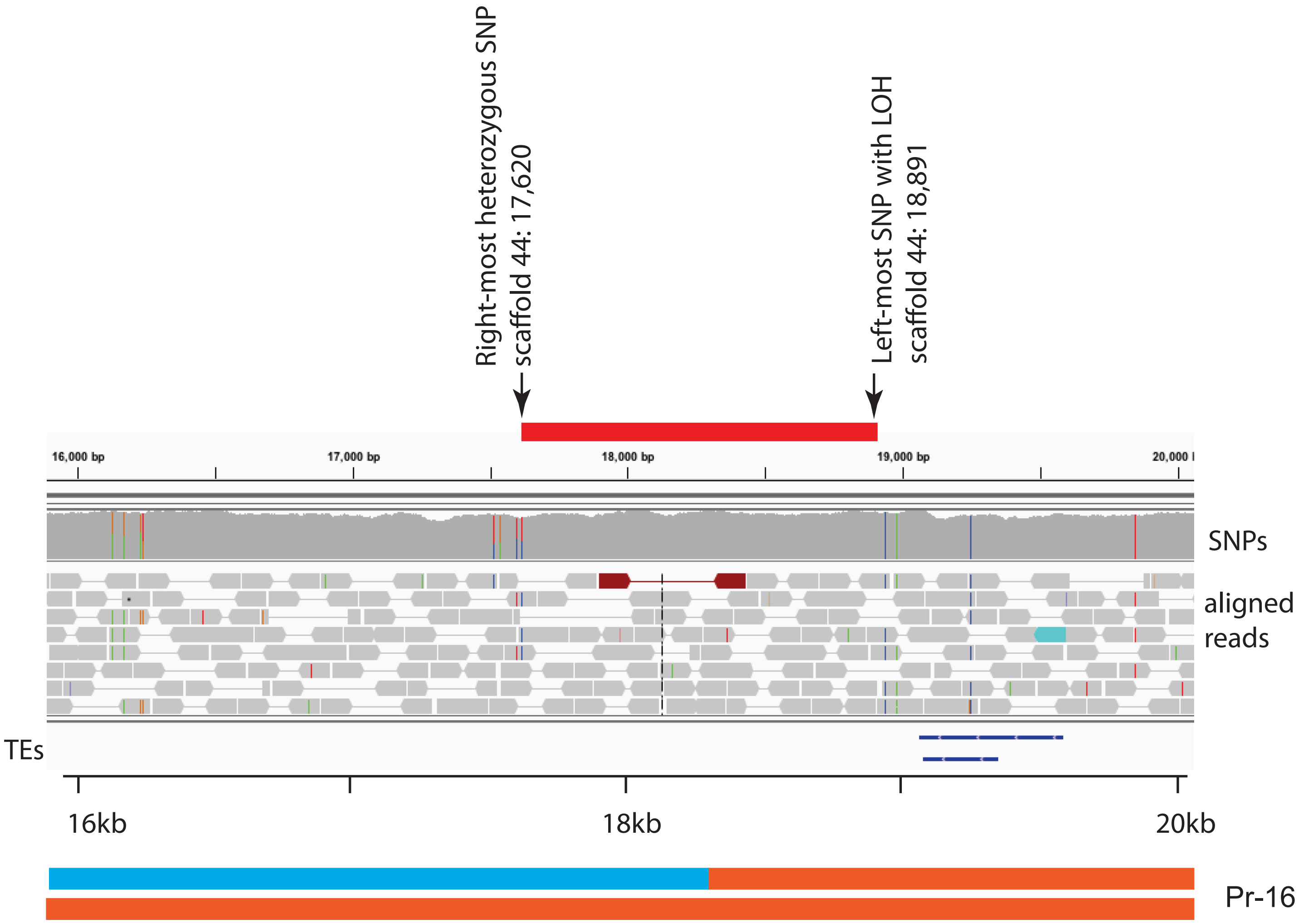

Additional file 7. The chromosomal breakpoint in scaffold 44. From the right most heterozygous SNP and the left most SNP with LOH, the breakpoint for Pr-16 (cnLOH) was inferred to be within the 1.3kb red rectangle region (top panel, Integrative Genomic Viewer ver. 2.3.34). Homologous chromosomes are depicted in orange and cyan (lower panel).
