# Supplementary material for: Host-induced aneuploidy and phenotypic diversification in the Sudden Oak Death pathogen Phytophthora ramorum
Source: BMC Genomics. 2016 May 20;17:385. doi: 10.1186/s12864-016-2717-z (PMC4875591; doi:10.1186/s12864-016-2717-z)
Supplement: Additional file 9: — Nwt colony morphology seen among EU1 isolates obtained from a single 4 m long lesion on a mature Lawson cypress. EU1 isolates (A-C) are derived from the top, (D-F) are from the middle, and (G-I) are from the bottom of the lesion. Mnwt and snwt indicate moderate and severe non-wild types, respectively. (PDF 234 kb) [file 12864_2016_2717_MOESM9_ESM.pdf]

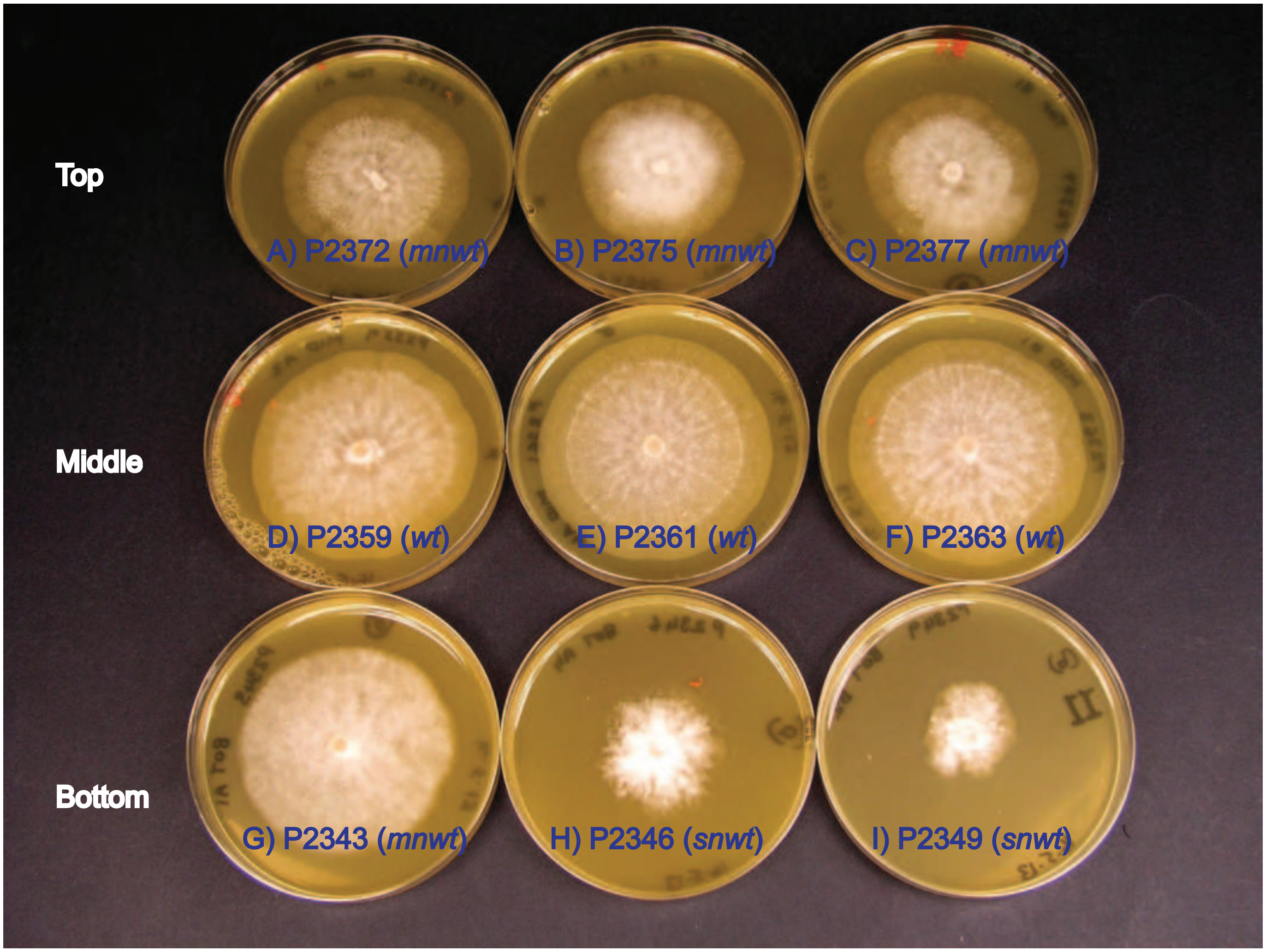

Additional file 8. *Nwt* colony morphology seen among EU1 isolates obtained from a single 4 meter long lesion on a mature Lawson cypress. EU1 isolates **(A-C)** are derived from the top, **(D-F)** are from the middle, and **(G-I)** are from the bottom of the lesion. *Mnwt* and *snwt* indicate moderate and severe non-wild types, respectively.
