# Supplementary material for: Host-induced aneuploidy and phenotypic diversification in the Sudden Oak Death pathogen Phytophthora ramorum
Source: BMC Genomics. 2016 May 20;17:385. doi: 10.1186/s12864-016-2717-z (PMC4875591; doi:10.1186/s12864-016-2717-z)
Supplement: Additional file 10: — BIC-seq analysis for three EU1 isolates using an NA1 isolate Pr-1556 (wt, bay) as a reference. Due to the genome divergence between the EU1 and NA1 lineages, a high background was seen. For an explanation of graphs, see Additional file 3. A) When the genome contents of EU1 wt isolate P2363 and the NA1 reference were compared, copy number reduction was detected for several regions (red bars), which accompany increase in SNP allele ratios, suggesting P2363 is a mixture of monosomic and disomic nuclei. B) When EU1 nwt isolate P2346 and the reference were compared, increases (red bars) as well as decreases in the genome content were detected. Note that several scaffolds show large heterozygous SNP ratios while copy number changes in corresponding scaffolds are subtle. This may implicate a formation of cnLOH. C) EU1 nwt isolate P2386 shows large SNP allele ratios in several scaffolds indicating the formation of cnLOH (red bars). These cnLOH scaffolds are, however, distinct from those observed in P2346. (PDF 561 kb) [file 12864_2016_2717_MOESM10_ESM.pdf]

A) P2363 (*wt, C. lawsoniana*) vs. Pr-1556 (*wt*, bay), CCNV heterokaryon

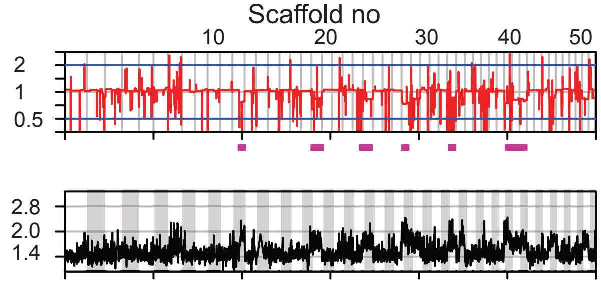

B) P2346 (*nwt, C. lawsoniana*) vs. Pr-1556 (*wt*, bay), CCNV heterokaryon,

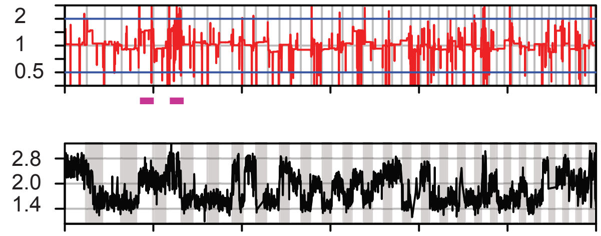

C) P2386 (*nwt, C. lawsoniana*) vs. Pr-1556 (*wt*, bay), CCNV heterokaryon

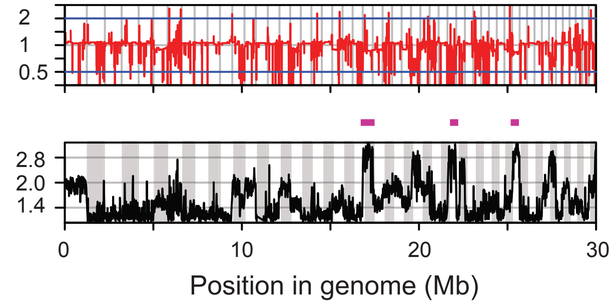

Additional file 9. BIC-seq analysis for three EU1 isolates using an NA1 isolate Pr-1556 (*wt*, bay) as a reference. Due to the genome divergence between the EU1 and NA1 lineages, a high background was seen. For an explanation of graphs, see Additional file 3. When the genome contents of EU1 *wt* isolate P2363 and the NA1 reference were compared, copy number reduction was detected for several regions (red bars), which accompany increase in SNP allele ratios, suggesting P2363 is a mixture of monosomic and disomic nuclei. B) When EU1 *nwt* isolate P2346 and the reference were compared, increases (red bars) as well as decreases in the genome content were detected. Note that several scaffolds show large heterozygous SNP ratios while copy number changes in corresponding scaffolds are subtle. This may implicate a formation of cnLOH. C) EU1 *nwt* isolate P2386 shows large SNP allele ratios in several scaffolds indicating formation of cnLOH (red bars). These cnLOH scaffolds are, however, distinct from those observed in P2346.
